# Transcriptional Landscapes of Long Non-coding RNAs and Alternative Splicing in *Pyricularia oryzae* Revealed by RNA-Seq

**DOI:** 10.3389/fpls.2021.723636

**Published:** 2021-09-08

**Authors:** Zhigang Li, Jun Yang, Junbo Peng, Zhihua Cheng, Xinsen Liu, Ziding Zhang, Vijai Bhadauria, Wensheng Zhao, You-Liang Peng

**Affiliations:** ^1^College of Plant Protection/Key Laboratory of Green Prevention and Control of Tropical Plant Diseases and Pests, Ministry of Education, Hainan University, Haikou, China; ^2^Ministry of Agriculture and Rural Affairs Key Laboratory of Pest Monitoring and Green Management, College of Plant Protection, China Agricultural University, Beijing, China; ^3^State Key Laboratory of Agrobiotechnology, College of Biological Sciences, China Agricultural University, Beijing, China

**Keywords:** rice blast fungus, conidiation, hyphal growth, gene differential expression, alternative splicing, long non-coding RNAs, premature termination codons

## Abstract

*Pyricularia oryzae* causes the rice blast, which is one of the most devastating crop diseases worldwide, and is a model fungal pathogen widely used for dissecting the molecular mechanisms underlying fungal virulence/pathogenicity. Although the whole genome sequence of *P. oryzae* is publicly available, its current transcriptomes remain incomplete, lacking the information on non-protein coding genes and alternative splicing. Here, we performed and analyzed RNA-Seq of conidia and hyphae, resulting in the identification of 3,374 novel genes. Interestingly, the vast majority of these novel genes likely transcribed long non-coding RNAs (lncRNAs), and most of them were localized in the intergenic regions. Notably, their expressions were concomitant with the transcription of neighboring genes thereof in conidia and hyphae. In addition, 2,358 genes were found to undergo alternative splicing events. Furthermore, we exemplified that a lncRNA was important for hyphal growth likely by regulating the neighboring protein-coding gene and that alternative splicing of the transcription factor gene *CON7* was required for appressorium formation. In summary, results from this study indicate that lncRNA transcripts and alternative splicing events are two important mechanisms for regulating the expression of genes important for conidiation, hyphal growth, and pathogenesis, and provide new insights into transcriptomes and gene regulation in the rice blast fungus.

## Introduction

*Pyricularia oryzae* (syn., *Magnaporthe oryzae*) is the fungal pathogen causing the rice blast disease, which has been threatening rice production worldwide. Globally, 10–30% of the annual rice harvest is lost due to the disease (Valent and Chumley, 1991; Talbot, 2003). *P. oryzae* is also the first sequenced plant pathogenic fungus (Dean et al., 2005) and has been widely used as a seminal model for investigating molecular mechanisms underlying pathogenesis in plant fungal pathogens (Valent and Chumley, 1991). Our group previously published the whole genome sequence of two field isolates of *P. oryzae* (Xue et al., 2012). The whole-genome sequencing of these strains in conjunction with recently released genome assemblies led to the prediction of approximately 13,000 protein-coding genes in *P. oryzae* (Dean et al., 2005; Xue et al., 2012; Gomez Luciano I, Tsai et al., 2019). However, 2,341 novel transcripts revealed by a recent RNA-Seq study were not included in the *P. oryzae* genome annotation (Kawahara et al., 2012). Expressed sequence tag (EST) analysis of nine cDNA libraries from seven different strains identified 134 genes that were undergone alternative splicing (Ebbole et al., 2004). In the latest annotation of the 70-15 strain, 155 genes were found to have multiple splicing isoforms. These studies indicate that our understanding of the *P. oryzae* genome is incomplete, and further systematic analyses are required to reveal the transcriptional landscape of the rice blast fungus.

As a systems-biology approach, transcriptome analysis provides a comprehensive understanding of orchestrated regulation of expressed genes of the genome. Transcriptome analysis is especially accelerated by the RNA sequencing (RNA-Seq) technology, which has been widely used for high throughput transcriptome analyses. RNA-Seq offers several advantages over other transcriptome analysis methods. Firstly, RNA-Seq is more sensitive and can detect a large dynamic range of expression levels. Secondly, it can be used to detect precise locations of transcription boundaries, sequence variations and alternative splicing events (Wang et al., 2009; Ozsolak and Milos, 2011). To date, RNA-Seq has been applied to transcriptome analyses of diverse organisms, including although not limited to fungi, such as *Saccharomyces cerevisiae*, *Schizosaccharomyces pombe*, *Aspergillus oryzae*, and *Fusarium graminearum* (Nagalakshmi et al., 2008; Wilhelm et al., 2008; Wang et al., 2010; Zhao et al., 2013). With wide applications of RNA-Seq, several important, novel regulatory mechanisms of gene expression were revealed. For example, it was found that most of the non-repetitive sequence in the budding yeast is transcribed and that there was heterogeneity in the 3′ end of transcripts of the same genes (Nagalakshmi et al., 2008). By determining both ends of the transcripts, it was found that most of the protein-coding genes in budding yeast express multiple isoforms, and over 26 major transcript isoforms were expressed for some ones (Pelechano et al., 2013). More interestingly, overlapping or even bicistronic transcripts could be generated between tandem genes (Pelechano et al., 2013).

In filamentous fungal transcriptomes, alternative splicing is a ubiquitous regulatory mechanism, although its frequency is not as high as in animals and plants (Kempken, 2013). In recent years, an increasing number of studies suggested its crucial roles in the survival and pathogenicity of fungal pathogens (Grützmann et al., 2014). Long non-coding RNAs (lncRNAs), as a type of non-coding RNAs with a length of over 200 nt, are another important regulatory mechanism but have been poorly understood for their functions in filamentous fungi. Several studies reported its significant roles in gene regulation. For instance, lncRNAs were important in the development of *F. graminearum* (Kim et al., 2018; Wang et al., 2021). LncRNA profiles during infection and development of *Ustilaginoidea virens* were elucidated (Tang et al., 2021). In *P. oryzae*, little is known about the comprehensive characteristics of alternative splicing and lncRNA profile.

In order to provide a global view of *P. oryzae* transcriptomes, we carried out comprehensive analyses on three sets of RNA-Seq data originating from conidia and hyphae of the two field isolates P131 and Y34. Annotation of the resulting transcriptome assemblies encompassed 3,374 new genes and 6,472 novel transcripts compared with the MG8 genome annotation. Interestingly, except for 6.8% encoding novel proteins, the vast majority of the newly identified genes were found to be expressed for lncRNAs, which might be involved in the regulation of conidiation and hyphal growth of *P. oryzae*. We also found that 2,358 genes had alternatively spliced events, an important mechanism to regulate gene expression. In addition, we evaluated the expression levels of individual genes and found that 2,108 genes were differentially expressed between conidia and hyphae. In general, our study provided new insights into the *P. oryzae* transcriptomes, which will be instrumental in understanding its functional genomes.

## Materials and Methods

### Sample Preparation

P131 and Y34, two field isolates of *P. oryzae* with available whole-genome sequence (Xue et al., 2012), were used in this study. Conidia were produced on oatmeal tomato agar (OTA) plates, as described previously (Peng and Shishiyama, 1988) and harvested from 7-day culture plates grown at 25°C under constant fluorescent light. Hyphae were collected from 2-day-old cultures in complete medium shaken at 150 rpm at 25°C. Total RNA was extracted from conidia and hyphae with the Trizol reagent (Invitrogen, United States) and then enriched by oligo (dT) beads (Qiagen) for cDNA synthesis, respectively. Reverse transcription was performed according to the manufacturer’s protocol (Invitrogen, United States). High-throughput cDNA libraries were prepared according to the Illumina whole transcriptome library preparation protocol and sequenced on the Illumina GA platform. This work was performed by the BGI company.

### Assembly of RNA-Seq Reads and Annotation

The transcriptome of *P. oryzae* was reconstructed using a hybrid assembly strategy that merges genome-guided and genome-independent assemblies (for details, see Supplementary Results). To annotate functions of the assembled transcripts, all were searched against the NCBI *nr* database (version 2013.02.25) using BLASTX (version 2.2.24) with an *E*-value cutoff of 10^–10^. OrfPredictor (Min et al., 2005) was employed to annotate ORFs. GO (Ashburner et al., 2000) and KEGG (Kanehisa et al., 2012) annotations were performed by online tools AgBase GOanna (McCarthy et al., 2006) and KAAS (Moriya et al., 2007), respectively. Subcellular localizations of proteins were predicted by WoLF PSORT program (Horton et al., 2007). RNA-Seq reads and gene structures were visualized by IGV v2.3 (Thorvaldsdottir et al., 2013).

### Detection of Isolate-Specific Transcripts

To detect transcripts absent in 70-15, the transcripts of P131 and Y34 assembled by genome-independent approach were aligned individually against the 70-15 genome by BLAT (Kent, 2002) and searched against proteins by BLASTX. A transcript with no hit or poor match (sequence identity < 90% and alignment coverage < 50% for BLAT, and *E*-value > 10^–10^ for BLASTX) in the genome sequence and proteins was considered to be absent in 70-15. To identify the isolate-specific transcripts, transcripts absent in 70-15 were compared with the genomes and proteins of P131 and Y34, released previously (Xue et al., 2012). The same criteria were used in the comparison.

### Comparison of Gene Expression

Raw reads of each sample were re-mapped onto the genome of 70-15 by Tophat (version 2.0.6). In this round of mapping, our new annotation was supplied to Tophat (with “–GTF”) as reference. Cuffdiff2 (version 2.0.2) was used to estimate gene expression and identify differentially expressed genes. FPKM was used to represent gene abundance. Option “–mask-file” was included in Cuffdiff2 to ignore reads that may come from tRNA and rRNA. A gene was considered to be differentially expressed between two conditions if the *q*-value (FDR-adjusted *p*-value) was ≤0.05.

### Analysis of Alternative Splicing

Alternative splicing events were classified into different patterns by the online tool AStalavista^1^ (Foissac and Sammeth, 2007). To assess the significance of variation between conidia and hyphae, a Fisher’s exact test was performed for each event. Fragment counts assigned to each isoform were estimated by Cufflinks, which were used in tests of significance. Fragments in the 2 × 2 table of Fisher’s exact test were divided into isoforms in an event on the one hand and into conidia and hyphae on the other hand. Fisher’s exact test *p*-value was adjusted by FDR. An event was designated as a variation between conidia and hyphae if it passed the FDR < 0.001 criterion. An analogous approach was performed to assess the significance of variation between P131 and Y34.

### Fungal Strains, Culture Conditions, Transformation, and Gene Deletion

Primer pairs uf/ur and df/dr were designed for amplifying ∼1.5 kb upstream and ∼1.5 kb downstream fragments from genomic DNA of the strain P131 and cloned into pKOV21 vector. The resulting vector was used to transform the P131 protoplasts to generate gene deletion mutants, as described previously (Kong et al., 2012). Transformants were selected via hygromycin resistance and then screened using the primer pairs ou/hu and od/hd. The resulting transformants were confirmed by Southern blot analysis of the genomic DNAs digested by *Xho*I and probed by the upstream fragment. The strain P131 and G003973 knockout transformants were cultured and maintained on the OTA plates (Peng and Shishiyama, 1988). Colony growth was assayed after incubation at 28°C for 5 days on OTA plates. Hyphae collected from complete medium were used for isolating fungal protoplasts that were used in transformation by the PEG/CaCl_2_ method (Park et al., 2004).

### Microscopy Assays

Appressorium formation on an artificial cover glass slide (hydrophobic surface) was assayed using conidia harvested with 2 mL sterilized distilled water from the 7-day-old OTA cultures, and appressorium formation rate was assessed at 24 h-post-inoculation (hpi), as described previously (Yang et al., 2012).

## Results

### Many RNA-Seq Reads Were Matched With Intergenic Regions and Predicted Introns in the *Pyricularia oryzae* (syn., *Magnaporthe oryzae*) Genome

In this study, three RNA samples originating from conidia (P131_conidia) and hyphae P131_hyphae of the P131 strain, and hyphae (Y34_hyphae) of the Y34 strain were sequenced onto Illumina GA, which resulted in the generation of 13,133,334, 48,125,694, and 47,846,570 paired-end reads, respectively (Supplementary Table 1).

**FIGURE 1:**
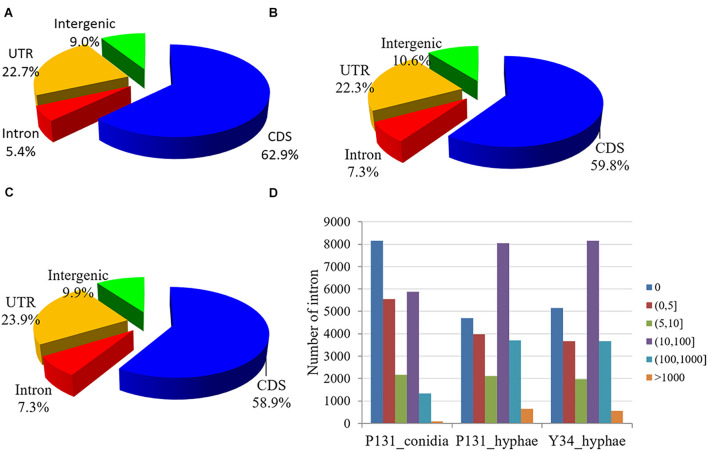
Read distribution in the genome. **(A–C)** Pie charts showing reads from the RNA-Seq data of P131_conidia **(A)**, P131_hyphae **(B)**, and Y34_hyphae **(C)** that mapped to the coding region (CDS), 5′- or 3′-untranslated regions (UTR), introns, and intergenic regions. **(D)** Read counts mapped onto the introns. Bins denote different groups of read counts. The Y-axis shows the number of introns.

When evaluated for read distribution over the genome, approximately 60 and 23% of the mapped reads from each sample matched to CDS and UTR, respectively. To our surprise, for each sample, ∼10% of the reads were mapped onto the intergenic regions, which is 5 and 3% higher than that in *Arabidopsis thaliana* (Filichkin et al., 2010) and *Mus musculus* (Mortazavi et al., 2008). Furthermore, 5.4, 7.3, and 7.3% of RNA-Seq reads from P131_conidia, P131_hyphae, and Y34_hyphae matched to the predicted introns, respectively (Figures 1A–C), which are 1.7, 1.4, and 4% higher than that in *F. graminearum* (Zhao et al., 2013), *A. thaliana* (Filichkin et al., 2010), and *M. musculus* (Mortazavi et al., 2008). Notably, more than half of the predicted introns were detected in RNA-Seq data (Figure 1D).

### Identification of 3,374 Novel Genes in *P. oryzae*

We generated a new annotation of the *P. oryzae* genome with a hybrid assembly strategy that integrates genome-guided and genome-independent methods for assembling the RNA-Seq reads, as shown in Supplementary Figure 1 (for details, see Supplementary Table 2 and Supplementary Results). This new annotation contains 16,192 genes (represented by 19,418 transcripts), 11,513 of which consist of multiple exons and the remaining 4,679 genes are single-exon genes. When compared with the MG8 annotation, our new annotation has 3,374 more genes, including 1,006 multiple exon genes and 2,368 single exon genes (Table 1). RNA-Seq data showed that at least 66% of the *P. oryzae* genome were expressed in hyphae and conidia, which was more than 54% of the MG8 annotation.

**TABLE 1:** Comparison between newly built annotation and the MG8 annotation.

	MG8 annotation	new annotation
**Genes**		
Number of genes	12,827	16,192
Number of genes + strand	6,423	7,179
Number of genes - strand	6,404	7,209
Number of genes no strand	0	1,804
Mean gene length (bp)	2,011	2,046
Number of genes with multiple isoforms	155	2,358
Number of genes with multiple exons	10,332	11,513
**Transcripts**		
Number of transcripts	12,991	19,418
Mean transcript length (bp)	1,802	1,928
Number of multiple exon transcripts	10,481	14,374

### Expression Profiles of Novel Protein-Coding Genes of *P. oryzae* Suggest Their Involvement in Hyphal Growth and Conidiation

**FIGURE 2:**
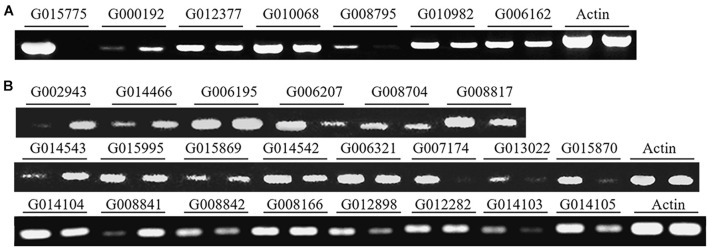
Validation of the transcripts of novel protein-coding genes and lncRNAs with RT-PCR. **(A)** Seven genes and **(B)** 22 lncRNAs were expressed in conidia (left lanes) and/or hyphae (right lanes). The actin gene was used as a control.

Among the 3,374 new genes identified in this study, 40 genes (including 10 of the 230 protein-coding genes) were randomly selected for RT-PCR validation. 29 of the genes, including seven of the protein-coding genes, were expressed in conidia and/or in hyphae (Figures 2A,B), suggesting that most of the newly identified protein-coding genes are expressed in *P. oryzae*.

**FIGURE 3:**
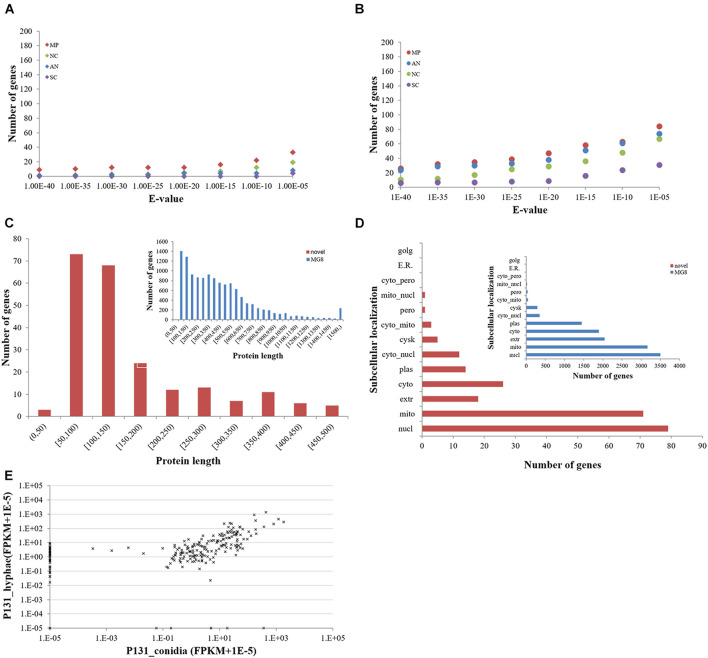
Features of novel protein-coding genes identified in this study. **(A)** The number of novel *Pyricularia oryzae* proteins with orthologs in other filamentous ascomycetes by BLASTN and **(B)** TBLASTN searches. MP, *Magnaporthe poae*; AN, *Aspergillus nidulans*; NC, *Neurospora crassa*; SC, *Saccharomyces cerevisiae*. **(C)** Length distribution and **(D)** subcellular localizations of novel *P. oryzae* proteins compared with those of proteins present in the MG8 annotation (inlets). **(E)** Expression levels of novel *P. oryzae* protein-coding genes (represented by FPKM) in conidia and hyphae.

To clarify if these protein-coding novel genes are conserved in other fungi, we searched them against the genomes of *M. poae*, *Aspergillus nidulans*, *Neurospora crassa*, and *S. cerevisiae* using BLASTN and TBLASTN (Figures 3A,B). To our surprise, only a small portion of the genes had orthologs in these four fungi, indicating that most of the newly identified protein-coding genes are unique to *P. oryzae*. Interestingly, 76 of the new *P. oryzae* genes were predicted to encode proteins with less than 100 amino acid residues (Figure 3C). When predicted for their subcellular localizations by WoLF PSORT, 79 of the new genes (34%) identified in this study encode putative nuclear proteins, which is the largest category (Figure 3D). Notably, 18 genes were predicted to encode extracellular proteins, including six proteins with less than 100 amino acid residues.

We also compared expression profiles of the novel protein-coding genes in conidia and hyphae. Thirty-eight were expressed only in hyphae, whereas six were specifically expressed in conidia (Figure 3E). Expression of 177 genes could be detected in both conidia and hyphae. Among them, 119 had more than 2-fold differences in expression levels between conidia and hyphae (47 higher in conidia and 72 higher in hyphae). These results indicate that many of the newly identified protein-coding genes in *P. oryzae* are involved in vegetative hyphal growth and/or conidiation.

With the genome-independent assembly, several hundreds of isolate-specific genes were identified in this study. In total, 270 and 358 assembled genes in P131 and Y34, respectively, were found to be absent in the genome of 70-15. Of these, 80 and 140 genes were found to be unique to P131 and Y34, respectively; 46 genes of P131, 78 genes of Y34 were novel genes identified in this study, and they lack homologous sequences in the *nr* database. Interestingly, 58 of the P131-specific genes (72.5%) and 109 of the Y34-specific genes (77.9%) were predicted to encode proteins smaller than 100 amino acid residues. More importantly, seven of the P131-specific genes encode extracellular proteins, three of which contain multiple cysteine residues. Five Y34-specific genes may also encode extracellular proteins. Two of them have multiple cysteine residues. These isolate-specific extracellular proteins with multiple cysteine residues may function as effectors that are important for pathotype differentiation in *P. oryzae*.

### Long Non-coding RNAs May Play Regulatory Roles in Conidiation and/or Hyphal Growth

**FIGURE 4:**
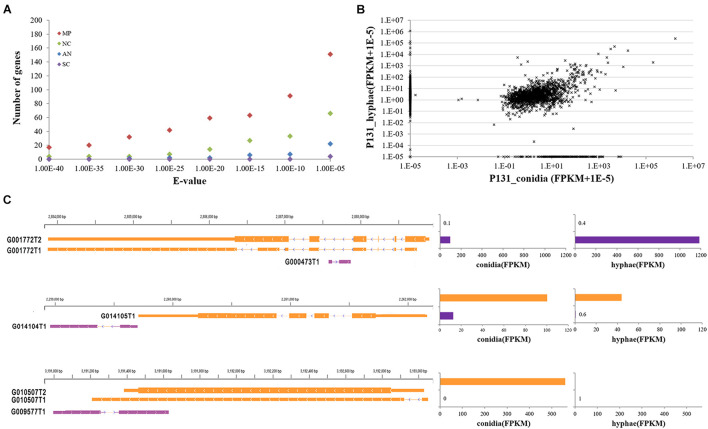
Features of *P. oryzae* lncRNAs. **(A)** The number of putative non-coding genes with hits in other fungi by BLASTN searches. MP, *Magnaporthe poae*; AN, *Aspergillus nidulans*; NC, *Neurospora crassa*; SC, *Saccharomyces cerevisiae*. **(B)** Expression levels of putative non-coding RNA transcripts in conidia and hyphae. Expression levels of each lncRNA are represented by FPKM. **(C)** Examples showing the expression of lncRNAs in relation to their neighboring genes, i.e., G001772 (MGG_16180), G014105 (MGG_04005), and G010507 (MGG_06585). The assembled transcript number for each gene was appended to gene id.

With the Coding Potential Calculator (CPC) that classifies transcripts into coding and non-coding groups based on six criteria (Kong et al., 2007), we identified 3,010 putative non-coding RNAs, most of which were putative species-specific genes (Figure 4A). Among them, 2,478 were transcribed from the intergenic regions, including 395 transcripts that were jointed from more than two exons. A total of 508 transcripts were transcribed from the antisense strand of protein-coding genes. Some of the antisense transcripts are putative intronic non-coding RNAs because they were transcribed from the introns of protein-coding genes, such as transcript G000473T1 mapped onto the intron of MGG_16180 (G001772). Notably, among the above-mentioned non-coding RNAs, 2,877 transcripts were longer than 200 nucleotides, including 1,515 longer than 500 nucleotides. Therefore, most of the non-coding RNAs identified in this study are lncRNAs. Among the 30 lncRNAs randomly selected for verification by RT-PCR, 22 were confirmed to be expressed in conidia and/or in hyphae (Figure 2B), confirming that most of the lncRNAs are transcribed in *P. oryzae*.

When examined for their transcript profiles, 216 and 859 non-protein-coding transcripts were found to be conidium- and hypha-specific, respectively (Figure 4B). In addition, 1,714 putative non-coding genes were expressed in both tissues. Among them, 449 and 618 had higher expression levels (≥2-fold changes) in conidia and hyphae. Notably, 29 and 14 lncRNAs were significantly up-regulated in conidia and hyphae, respectively (*q*-value ≤ 0.05). These results suggested that some of the lncRNAs are differentially regulated in conidia and vegetative hyphae.

We further checked the expression levels of 2,773 lncRNAs in relation to their neighboring protein-coding genes in conidia and hyphae. The expression of 1,631 lncRNAs was found to be associated with the expression of their neighboring protein-coding genes (Figure 4C), suggesting that these lncRNAs may be involved in regulating expression of their neighboring genes. For example, G014104T1 is a 552-nt lncRNA originating from an intergenic region downstream of MGG_04005, which was expressed at 13 FPKM in conidia and 0.6 FPKM in hyphae. MGG_04005 was expressed consistently at 100 FPKM in conidia and 44 FPKM in hyphae, respectively. Another example is MGG_16180, which was annotated with two alternatively spliced transcripts (i.e., G001772T1 and G001772T2) and was expressed at 0.1 and 0.4 FPKM in conidia and hyphae. The intronic lncRNA, G000473T1, was expressed at 96.8 and 1187.22 FPKM in conidia and hyphae (Figure 4C). The expression relationship of these lncRNAs with their neighboring genes was verified by quantitative RT-PCR (Supplementary Figure 2).

**FIGURE 5:**
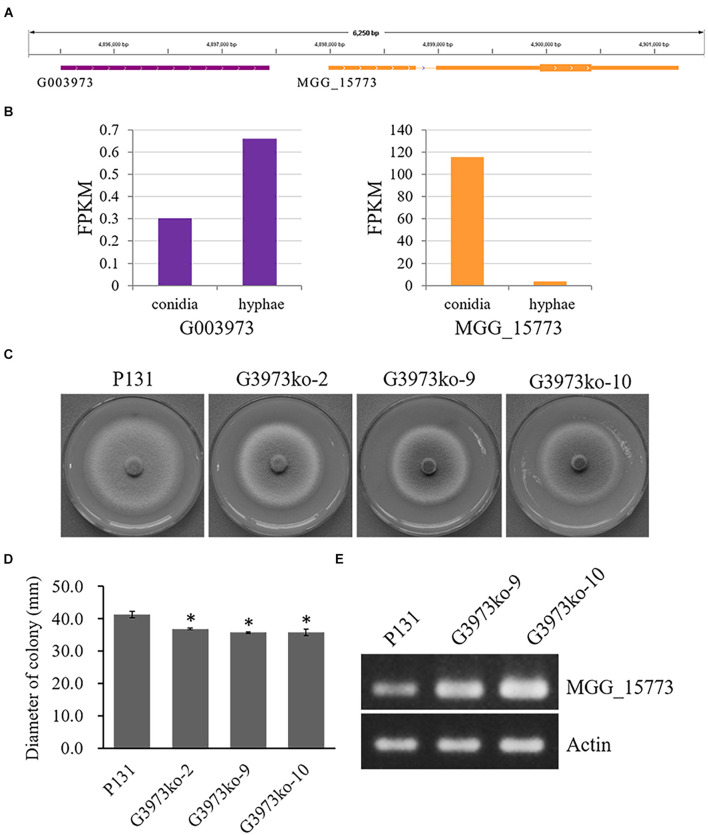
The G003973 lncRNA is important for colony growth. **(A)** The G003973 lncRNA was 544 bp upstream from MGG_15773. **(B)** The expression levels of G003973 and MGG_15773 in conidia and hyphae, respectively. **(C)** Colonies of the wild-type strain P131, the G003973 deletion mutants G3973ko-2, G3973ko-9, and G3973ko-10 on OTA plates at 5 dpi. **(D)** Bar graphs showing the diameters of colonies P131, G3973ko-9, and G3973ko-10 on OTA plates at 5 dpi. Means ± SE were calculated from three independent replicates. ^∗^*P* < 0.01 (Student’s *t*-test). **(E)** Expression levels of MGG_15773 in strains P131, G3973ko-9, and G3973ko-10, and the actin gene was used as a control.

These data indicate that most of the lncRNA genes identified in this study may be involved in the regulation of conidiation and/or hyphal growth. Many of these lncRNA may function to enhance the expression of their putative target genes, whereas many others can suppress the expression of their putative target genes. To verify the regulatory role of lncRNAs, we generated three knockout mutants of one lncRNA, G003973T1 (Supplementary Figure 3), which may be important for hyphal growth and negatively regulate the expression of MGG_15773 as suggested by the RNA-seq data (Figures 5A,B). All the three deletion mutants formed similarly smaller colonies as compared with the wild-type strain P131 (Figure 5C), indicating that G003973T1 positively regulates hyphal growth. We further measured expression levels of MGG_15773 in two of the knockout mutants. As shown in Figure 5D, the knockout mutants showed significantly higher expression of MGG_15773 than the wild-type P131 in hyphae, verifying that G003973T1 negatively regulates the expression of MGG_15773. Together, these results indicated that the lncRNA G003973T1 is important for hyphal growth and negatively regulates expression of its neighboring gene MGG_15773.

### *Pyricularia oryzae* Has Thousands of Genes That Express Multiple Forms of Transcripts

**FIGURE 6:**
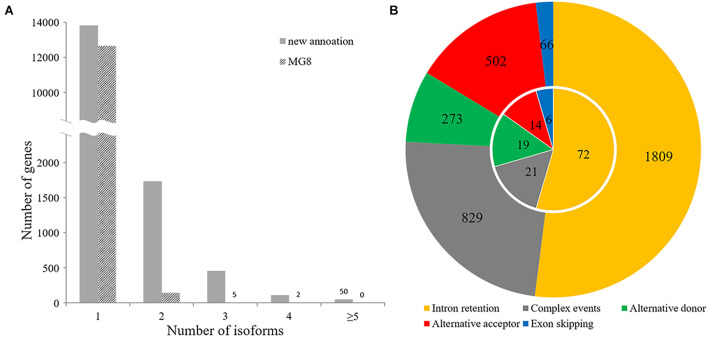
Alternative splicing analysis. **(A)** Distribution of the number of transcript isoforms per gene in the incumbent annotation and MG8 annotation. **(B)** Circle plot of patterns of alternative splicing events. The inner and outer circles indicate alternative splicing events observed in the MG8 annotation (strain 70-15) and this annotation.

Our annotation showed that 2,358 of 16,192 *P. oryzae* genes belonging to various function categories (Supplementary Table 3) had multiple forms of transcripts, including dozens of genes known to be important or essential for pathogenicity, such as *HEX1* and *MoPPG1* (Soundararajan et al., 2004; Du et al., 2013). A total of 1,738 genes had two forms of transcripts and 620 had more than three isoforms (Figure 6A), indicating that alternative splicing frequently occurs in many genes of the rice blast fungus.

The 3,479 alternative splicing events identified with AStalavista (Foissac and Sammeth, 2007) in 2,045 genes could be classified into different alternative splicing patterns, including alternative donor, alternative acceptor, exon skipping, intron retention, and “complex events” (Figure 6B). Among them, 1,809 belonged to intron retention, which accounted for approximately 52% of the total alternative splicing events, suggesting that intron retention is the most prevalent alternative splicing in *P. oryzae*. Whereas 502 alternative acceptor events (14%) and 273 alternative donor events (8%) were identified, we observed only 66 exon skipping events. In addition, about one-quarter of alternative splicing events were classified as “complex events” that may involve two or more common splicing patterns.

To reveal whether alternative splicing is differentially regulated between conidia and hyphae, a Fisher’s exact test (Wang et al., 2008) was performed to compare expression levels of alternatively spliced transcripts (see Section “Materials and Methods”). A total of 1,593 alternatively spliced transcripts were found to have significant differences in abundance between conidia and hyphae of P131. The alternative splicing event involved in these transcripts and functions of the corresponding genes were shown in Supplementary Figure 4. 1,417 alternatively spliced transcripts from 651 genes were found to contain the same open reading frames. Notably, intron retention was identified in UTR regions of 393 transcripts. Furthermore, 701 alternatively spliced transcripts of 1,417 were significantly differentially expressed between conidia and hyphae.

**FIGURE 7:**
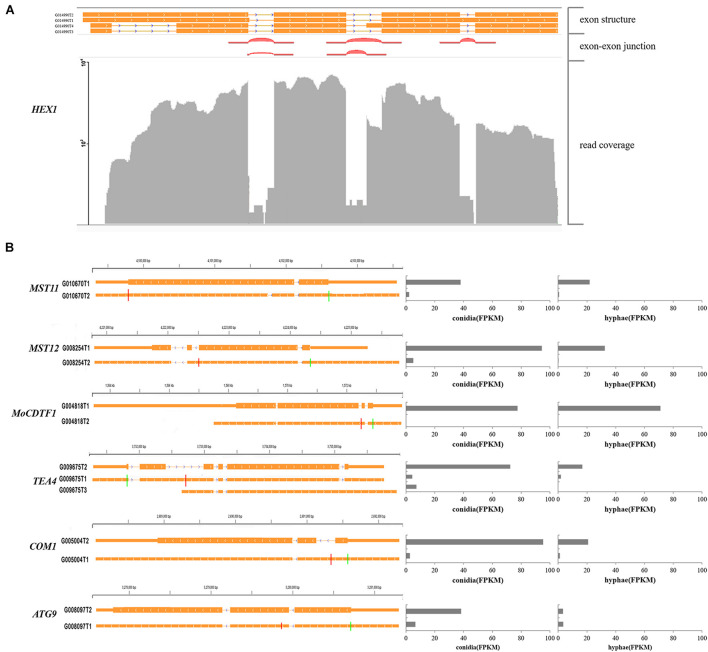
Examples of pathogenicity-related genes with multiple transcripts. **(A)** Alternative transcripts of *HEX1*. Exon-exon junctions were determined by Tophat. **(B)** Alternative transcripts (left) and their expression levels (right) of the marked pathogenicity-related genes. For each gene, a line meant a transcript of the gene. Exons are illustrated as orange lines, and the thick regions in exons indicate the coding regions. The green and red vertical lines represent translation start and stop sites, respectively. Chromosome coordinates are shown above each gene.

Furthermore, we identified 537 alternatively spliced transcripts from 249 genes that shared the same start/stop codons but encoded distinct proteins. Of these transcripts, 230 alternative transcripts differed significantly in their abundance between conidia and hyphae. Several previously characterized genes were found to have this type of alternative splicing. For instance, the *HEX1* gene that is essential for full virulence and survival under nitrogen starvation conditions was found to have four alternatively spliced transcripts. Two of them that were described in a previous study (Soundararajan et al., 2004) encode a 439- and a 417-aa protein, respectively (Figure 7A). The *MST11* (MGG_14847) gene (Zhao et al., 2005) had two transcripts. Whereas G010270T2 encodes an 894-aa protein, G010670T1 encodes a 915-aa protein. The latter retained a 63-bp intron and was more abundant in both conidia and hyphae (Figure 7B).

To our surprise, 2,112 alternatively spliced transcripts from 938 genes contained premature termination codon (PTC), including 957 transcripts of the 453 genes that had a significant difference in their expression levels between conidia and hyphae. We compared the expression levels of the PTC transcripts with their non-PTC transcripts. Interestingly, 666 PTC transcripts were less abundant in both conidia and hyphae than the normal transcripts from the same genes. Additional 232 PTC transcripts were less abundant either in conidia or in hyphae than the normal transcripts. Furthermore, 21 and 64 transcripts with PTC were found to be conidium- and hypha-specific, respectively. GO term enrichment analysis showed that 393 of those genes with PTC transcripts encode proteins with binding activities, i.e., protein binding, nucleic acid binding and ion binding (Supplementary Table 4). The other functions included catalytic activity (352 genes), transporter activity (87 genes), enzyme regulator activity (51 genes), and signal transducer activity (19 genes) (Supplementary Table 4).

**FIGURE 8:**
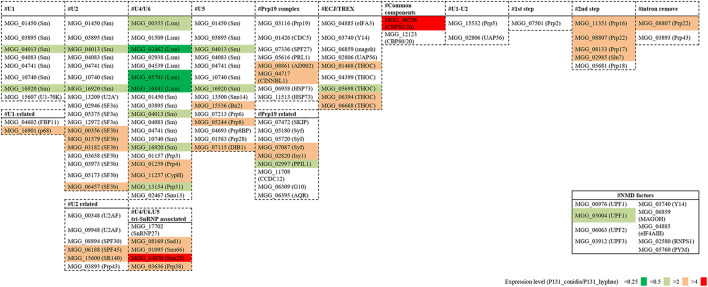
Fold changes in expression levels of splicing factor genes and NMD pathway genes between the conidia and the hyphae.

To understand how the basal splicing machinery is involved in the above-mentioned alternative splicing, we analyzed the expression levels of 109 conserved components of the spliceosome (Kanehisa et al., 2012). For more than 60 of them, their expression levels were similar in conidia and hyphae (Figure 8). However, about 40 of the genes had increased or decreased expression levels in conidia, suggesting that differential expression of some components of the spliceosome may be important for the alternative splicing. For example, SF3b1 (MGG_00356), which is essential for intron definition in yeast (Shao et al., 2012), was much more expressed in conidia than in hyphae (Supplementary Figure 5A). We also compared the expression levels of genes essential for nonsense-mediated decay (NMD; Chang et al., 2007; Kanehisa et al., 2012), and observed that *UPF1* (MGG_03004) was much more expressed in hyphae than in conidia (Supplementary Figure 5B) although almost all the other NMD-related genes had similar expression levels in conidia and hyphae (Figure 8).

### Alternative Splicing Events of Genes Known to Be Important for Hyphal Growth, Conidiation, and Pathogenesis

In our annotation, transcripts from many previously characterized genes were found with alternative splicing events. For example, *MST12* (MGG_12958; Park et al., 2002) were found to have two alternatively spliced transcripts, one of which was 4,027 bp in length for a protein with 715 amino acid residues as previously reported and was accumulated at 94 FPKM in conidia and 32 FPKM in hyphae. The other form was 4,651 bp in length with a 111 bp intron but encoded a truncated protein with 583 amino acids and was accumulated at 5 FPKM in conidia and 0.3 FPKM in hyphae (Figure 7B), suggesting that PTC transcripts of *MST12* were less abundant. Another example was *MoCDTF1* (MGG_11346; Yan et al., 2011) annotated with two transcripts. In comparison with the normal transcript, the other transcript had an intron retention that led to premature termination. The aberrant transcript was less abundant in both conidia and hyphae than the normal transcript of *MoCDTF1* (Figure 7B). These PTC transcripts may subsequently become targets for NMD, an RNA surveillance mechanism that recognized mRNAs containing PTCs and degrades them, and were less abundant (Maquat, 2004).

Some genes that were differentially expressed between conidia and hyphae had aberrant transcripts as well. For instance, *TEA4* (MGG_06439) is important for conidiation (Patkar et al., 2010). Among the transcripts identified in this study, the normal transcript encoding an 812-aa protein was highly accumulated in conidia, whereas the transcript with the retention of the second intron (579 bp) encoding a truncated 263-aa protein was less abundant. The *COM1* gene (MGG_01215) required for normal conidial morphology (Yang et al., 2010) also had two transcripts. Compared to the normal transcript that was much more abundant in both conidia and hyphae, the alternative transcript retained the first intron (266 bp) and encoded a truncated 66-aa protein. We also found that *ATG9* (MGG_09559) (Kershaw and Talbot, 2009) had two transcripts. The normal transcript encoding a 917-aa protein was highly accumulated in conidia. Both normal transcript and the alternative transcript encoding a 246-aa protein resulted from an alternative donor for the first intron were expressed equally in hyphae.

**FIGURE 9:**
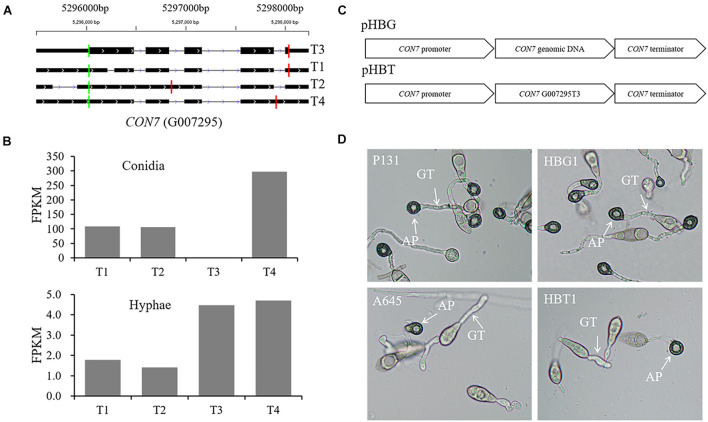
Functional characterization of alternatively spliced transcripts of *CON7*. **(A)** Diagram of four different transcripts of *CON7*, T1, T2, T3 (=MGG_05287T0), and T4. Each line meant a transcript of *CON7*. Exons are illustrated as black lines, and the thick regions in exons indicate the coding regions. The green and red vertical lines represent translation start and stop sites, respectively. **(B)** Bar graphs showing the expression levels of the four *CON7* transcripts (T1, T2, T3, and T4) in conidia and hyphae. **(C)** Schematic diagram of pHBG and pHBT vectors. pHBG contains the genomic DNA fragment of *CON7*. pHBT contains the transcript G007295T3. pHBG and pHBT were transformed into the A645. **(D)** Appressorium formation on hydrophobic cover glass slides with conidia of the wild-type strain P131, the *con7* insertion mutant A645, strains pHBG and pHBT at 12 hpi. AP, appressorium; GT, germination tube.

*CON7* encodes a transcription factor Con7p that regulates multiple phenotypes, including appressorium formation (Odenbach et al., 2007; Cao et al., 2016). In this study, we identified four *CON7* transcripts (Figure 9A), including transcript G007295T3 that is annotated as the functional form encoding a 412-aa protein.^2^ Transcripts G007295T2 and G007295T4 were the two transcripts with alternative splicing events. These four transcripts were differentially expressed between conidia and hyphae (Figure 9B). To investigate whether the alternative splicing transcripts are important for appressorium formation, we constructed two vectors, pHBG and pHBT, in which pHBG contained the genomic region of the entire *CON7* gene and pHBT had the transcript G007295T3 fragment under the control of the *CON7* promoter (Figure 9C). These two constructs were individually introduced into the *con7* mutant A645 (Kong et al., 2013), and over 10 independent transformants were obtained for phenotypic analysis. To our surprise, none of the pHBT transformants rescued appressorium formation, while all the pHBG transformants were normal in appressorium formation (Figure 9D). These results indicated that the current annotated *CON7* transcript is not enough to rescue appressorium formation, but suggested that the alternative splicing of *CON7* is important for regulating appressorium formation.

### Many Genes May Be Differentially Expressed Between Conidia and Hyphae

Among the 16,192 annotated genes, 2,108 of them were found likely to be differentially expressed between conidia and hyphae. The expression of 664 genes was up-regulated in conidia, including 291 and 373 genes that were highly and specifically expressed (>1 FPKM) in conidia, respectively. In contrast, 1,444 genes had induced expression in hyphae, including 302 and 1,142 genes that were highly and specifically expressed in hyphae (>1 FPKM), respectively (Supplementary Table 5, Supplementary Figures 6–8, and Supplementary Results for further analyses on gene quantification).

Several genes known to be important for conidial development were highly expressed in conidia, including *ACR1* (MGG_09847; Lau and Hamer, 1998), *MoFLP1* (MGG_02884; Liu et al., 2009), and *MoCON6* (MGG_02246; Madi et al., 1994). In addition, many genes involved in pathogenicity were differentially expressed between conidia and hyphae as well. For example, the following genes were specifically up-regulated in conidia: *MoSFL1* (MGG_06971; Li et al., 2011), *ICL1* (MGG_04895; Wang et al., 2003), *GAS1* (MGG_12337; (Xue et al., 2002), *SLP1* (MGG_10097; Mentlak et al., 2012; Chen et al., 2014), *PTH11* (MGG_05871; DeZwaan et al., 1999), and *ERL1* (MGG_02549; Heupel et al., 2010). In contrast, *PTH3* (MGG_07528; Sweigard et al., 1998), *MgSM1* (MGG_05344; Jeong et al., 2007; Oh et al., 2008), *OMO1* (MGG_04212; Hof et al., 2007) were significantly induced in hyphae. We confirmed that the chitin synthase genes *CHS*1, *CHS*3, and *CHS*7 were more expressed in conidia as previously reported (Kong et al., 2012), Notably, some genes implicated in chitin degradation were likely to be up-regulated in conidia (Supplementary Figure 9 and Supplementary Results for further analyses on gene quantification).

## Discussion

In this study, we generated a new annotation for the *P. oryzae* genome by assembling three sets of RNA-Seq data that originated from conidia and hyphae of the two field isolates P131 and Y34 (Xue et al., 2012). In comparison with the MG8 annotation, our annotation includes 3,374 more genes and 6,427 novel transcripts, demonstrating the feasibility of building gene models of *P. oryzae* with RNA-Seq data. RNA-Seq is more sensitive and accurate than the automatic annotation procedures, which use *ab initio* gene predictions and sequence similarity searches. To construct the transcriptomes of *P. oryzae*, we used a hybrid assembly approach that integrated genome-guided and genome-independent methods, which may work better for capturing both known and novel variations (Garber et al., 2011). As a result, several thousands of alternatively spliced transcripts were identified in this study. Furthermore, many isolate-specific genes were identified with the genome-independent assembly. In general, our data showed that *P. oryzae* has more expressed loci and more isoforms of transcripts than previously reported. To better understand the *P. oryzae* transcriptomes, further investigation will be required to analyze RNA-Seq data from appressoria, *in planta* infection stages and diverse environmental stimuli, and to define transcription boundaries of transcripts, which have been reported to be highly divergent in the *S. cerevisiae* transcriptomes (Pelechano et al., 2013).

One important discovery in this study is the identification of approximately 3,000 expressed loci that have not been previously reported in *P. oryzae*. Most of these loci lie in the intergenic regions, many are in introns or antisense strands of protein-coding genes. Notably, many of the loci are expressed likely as lncRNAs, and only a few of them have homologs in other organisms (Figure 4A). Therefore, variation of lncRNAs might have undergone rapid adaptive selection in *P. oryzae*, as reported in other organisms (Pang et al., 2006). Thousands of lncRNAs have been discovered in mammal genomes (Nakaya et al., 2007; Guttman et al., 2009; Hangauer et al., 2013), and some amount of lncRNAs have also been identified in plants (Heo and Sung, 2011). There have been also some case studies on the functions of lncRNAs in the budding yeast. For instance, transcription of two lncRNAs (*IRT1* and *IME4-AS*) confers mating-type regulation of gametogenesis (van Werven et al., 2012). Although a broad functional repertoire of lncRNAs in other organisms has been studied (Mercer et al., 2009), their functions in *P. oryzae* have not been reported yet.

This study identified that there existed difference between conidia and hyphae in the expression of lncRNAs, suggesting they may play regulatory roles in conidiation and hyphal growth. Many of the intergenic lncRNA transcripts are likely expressed in association with their neighboring genes (Figure 4C), suggesting they may regulate the expression of the neighboring genes. As an example, we showed that deletion of a lncRNA locus led to the formation of smaller colonies and increased expression of its neighboring gene (Figure 5). These data clearly indicate that lncRNAs play important regulatory roles in conidiation and hyphal growth of *P. oryzae*. Further studies will be required to define functional roles of individual lncRNAs in the growth, asexual developments and pathogenesis of *P. oryzae*.

In this study, a total of 2,358 genes were found to have multiple isoforms of transcripts, which accounted for nearly 15% of 16,192 predicted genes, indicating that alternative splicing may be a global regulatory mechanism to enrich the functional repertoire of *P. oryzae*. Similarly, 15.4% (2,077 genes out of 13,487) of *Aspergillus flavus* genes were predicted to undergo alternative splicing (Chang and Muddiman, 2011). In contrast, only 1.3–4.2% of genes have alternative splicing in *N. crassa* and *F. graminearum* (Kempken, 2013; Zhao et al., 2013). Therefore, alternative splicing is a common regulatory mechanism in fungi, but its prevalence varies considerably among different fungi. However, without comparing RNA-Seq data extensively, we may overlook the differences.

Alternative splicing occurs in diverse processes of eukaryotes. For instance, it is important for meiosis (Engebrecht et al., 1991) and responses to environmental stress (Pleiss et al., 2007). In the plant pathogenic oomycete *Pseudoperonospora cubensis*, a multidrug resistance transporter gene is reported to encode a functional effector by an alternative splicing event (Savory et al., 2012). Especially, expression levels of alternative isoforms are regulated in different tissues or in different growth conditions (Wang et al., 2008). Our study explored the genome-wide alternative splicing events in the conidia and hyphae of *P. oryzae*, and showed that about half of the alternatively spliced transcripts varied significantly between conidia and hyphae. Notably, diverse functional genes have multiple alternatively spliced transcripts, including transcription factors, RNA recognitions and protein kinases (Supplementary Figure 4E). Therefore, we speculate that alternative splicing takes place widely in *P. oryzae* as an important mechanism to regulate conidiation and hyphal growth, regulate growth, asexual developments, pathogenesis and responses to environmental stimuli. It is well known that distinct functional proteins can be produced from a single gene by alternative splicing. For instance, two functionally distinct proteins are generated from the *PTC7* gene in many fungi (Marshall et al., 2013). In this study, we showed that 249 genes had multiple forms of transcripts that encode distinct proteins, including *HEX1*. Among them, alternative transcripts in 109 genes were differentially expressed between conidia and hyphae, including *MST11*. Notably, 10 and 22 alternative transcripts were conidium-specific and hyphae-specific, respectively. It will be important to determine what roles are played by these distinct forms of proteins from the same genes.

We further showed that *CON7*, a transcription factor gene essential for appressorium formation (Odenbach et al., 2007; Kong et al., 2013), had four types of transcripts, including two PTC forms. However, the previously annotated transcript encoding the longest protein failed to rescue the defect of the *con7* disruption mutant (Figure 9). Accumulation of PTC transcripts has not yet been reported in filamentous fungi, but it has been recognized as an important mechanism to regulate gene expression for normal granulocyte differentiation (Wong et al., 2013). The PTC transcripts are usually generated by intron retention, and their accumulation may activate the NMD pathway, which has been reported to be essential for the normal development of hematopoietic stem and progenitor cells in mammalian organisms (Weischenfeldt et al., 2008). However, the impact of NMD and PTC remains to be explored in filamentous fungi. Taken together, alternative splicing, an essential mechanism shaping the functional complexity of gene expression, plays a crucial role in conidiation, hyphal growth, and pathogenesis in filamentous fungi. Regulation of alternative splicing is a sophisticated process. A number of components and molecular features were involved, e.g., *cis*-acting elements and trans-acting factors, chromatin structure and RNA structure. Alternative splicing RNA with distinct, alternative transcription initiation or PTC generated genetic diversity as well (Wang et al., 2015).

Approximately 230 genes of the newly identified *P. oryzae* genes in this study may encode proteins. In addition, 80 P131-unique and 140 Y34-unique genes were also identified based on genome-independent assembly. Half of them were not annotated previously (Xue et al., 2012). Interestingly, most of these genes encode small proteins. Some of them were predicted to encode extracellular proteins that are rich in cysteine residues, indicating that they may function as effector proteins (Stergiopoulos and de Wit, 2009). The roles of these genes in vegetative growth, conidiation and pathogenesis require functional characterization thereof.

## Conclusion

In this study, we adopted a hybrid approach that integrated genome-guided and genome-independent assemblies of the RNA-seq data to construct the transcriptomes of *P. oryzae* in conidia and hyphae. As a result, a new annotation for *P. oryzae* was contrived and contained 16,192 genes with 19,418 transcripts, including 3,347 previously unknown new genes and 6,472 novel transcripts. Notably, the vast majority of the newly identified genes were found to be transcribed in conjunction with lncRNAs, and approximately 2,000 genes had alternative splicing events. Furthermore, we revealed that thousands of genes were differentially expressed between conidia and hyphae of *P. oryzae* and that lncRNAs and alternative splicing are two important mechanisms to regulate gene expression in *P. oryzae*. In summary, our analyses provided a deeper insight into lncRNAs and alternative splicing, which are involved in the regulation of development- and infection-related genes in *P. oryzae*.

## Data Availability Statement

The datasets presented in this study can be found in online repositories. The names of the repository/repositories and accession number(s) can be found here: NCBI PRJNA747097.

## Author Contributions

Y-LP and JY planned and designed the research. JP, ZC, and XL performed the experiments. ZL, JY, WZ, Y-LP, and ZZ analyzed the data. ZL, JY, Y-LP, VB, and XL wrote the manuscript. All authors contributed to the article and approved the submitted version.

## Conflict of Interest

The authors declare that the research was conducted in the absence of any commercial or financial relationships that could be construed as a potential conflict of interest.

## Publisher’s Note

All claims expressed in this article are solely those of the authors and do not necessarily represent those of their affiliated organizations, or those of the publisher, the editors and the reviewers. Any product that may be evaluated in this article, or claim that may be made by its manufacturer, is not guaranteed or endorsed by the publisher.

## Footnotes

1
http://genome.crg.es/astalavista/

2
https://www.broadinstitute.org/scientific-community/science/projects/fungal-genome-initiative/magnaporthe-comparative-genomics-proj

## Supplementary Material

Supplementary Figure 1 Pipeline showing assembly strategy. A hybrid strategy is employed to assemble RNA-Seq data from three samples. This strategy includes three steps: genome-independent assembly, genome-guided assembly and a combination of assemblies. Boxes with brown backgrounds indicate data sets, boxes with white background mean processing procedures, and boxes with broken green frames are original data used in the assemblies.

Supplementary Figure 2 Relative expression levels of different transcripts. Bar graphs showed the relative expressions of **(A)** G000473 and G001772 (MGG_16180), **(B)** G014104 and G014105 (MGG_04005), **(C)** G009577 and G010507 (MGG_06585), and **(D)** G003973 and G003974 (MGG_15773) in mycelium and conidia with quantitative RT-PCR assays, respectively.

Supplementary Figure 3 Gene knockout of G003973 lncRNA and validation. **(A)** G003973 and its gene replacement vector (constructed by replacing G003973 with *hph*). The position of primers ou, od, uf, ur, df, dr, hu, and hd are indicated with small arrows. X, *Xho*I. **(B)** DNA gel blot on *Xho*I-digested DNAs of strains P131 and the G003973 deletion mutants G3973-2, G3973-9, and G3973-10 hybridizing against probe 1 in panel **(A)**. The estimated sizes of each band are marked at right.

Supplementary Figure 4 Comparison of inter-tissue and inter-isolate alternative splicing variations. **(A,B)** The extent of alternative splicing events that are significantly regulated between tissues and isolates. **(C,D)** GO categories of genes with significantly regulated alternative splicing events between tissues and isolates. **(E)** Pfam domains of genes with significantly regulated alternative splicing events between tissues.

Supplementary Figure 5 Relative expression levels of different genes. Bar graphs showed the relative expression levels of **(A)** MGG_00356 and **(B)** MGG_03004 in hyphae and conidia, respectively.

Supplementary Figure 6 Overall comparisons of gene abundances. **(A,B)** are pair-wise scatterplots of gene expressions. The scatterplot indicates the global change of gene abundances between the two samples. Gene abundances are represented by FPKM. **(C)** Overall similarities between the samples are indicated by the clustering based on Euclidean distances. **(D)** Venn diagram indicating expressed genes in the three samples. Number in each set means gene number.

Supplementary Figure 7 Volcano plots depicting the relationship between fold-change and significance. Each point represents a gene in our new annotation. Red points indicate that their expression levels are significantly different between samples. **(A)** Comparison between P131_hyphae and P131_conidia**. (B)** Comparison between Y34_hyphae and P131_hyphae.

Supplementary Figure 8 Differentially expressed (DE) genes between P131 conidia and P131 hyphae. **(A)** Bar graph depicts subcellular localizations of proteins encoded by DE genes. **(B)** GO categories of DE gene-encoded proteins. **(C)** Pfam categories of highly expressed genes in conidia or hyphae. **(D–G)** Log_2_ (fold change) of highly expressed genes annotated as **(D)** transcription factors, **(E)** chitin-binding proteins, **(F)** sugar transporters, and **(G)** G-protein coupled receptors.

Supplementary Figure 9 Regulations of chitin synthesis and degradation. **(A)** Pathways of chitin synthesis and degradation. Genes represented by red boxes are up-regulated in conidia compared with hyphae. **(B–F)** Comparison of gene expression levels of *CHS*, chitinases, beta-*N*-acetyl hexosaminidases, chitin deacetylase and exo-1,4-beta-*D*-glucosaminidase in conidia and hyphae.

Supplementary Table 1 Summary of RNA-Seq data.

Supplementary Table 2 Summary of assemblies.

Supplementary Table 3 Functional categories of genes with multiple isoforms.

Supplementary Table 4 Functions of genes with alternative splicing.

Supplementary Table 5 Functions of genes differentially expressed between conidia and hyphae.
