# Supplementary figures and images for: Transcriptional Landscapes of Long Non-coding RNAs and Alternative Splicing in Pyricularia oryzae Revealed by RNA-Seq
Source: Front Plant Sci. 2021 Sep 8;12:723636. doi: 10.3389/fpls.2021.723636 (PMC8475275; doi:10.3389/fpls.2021.723636)

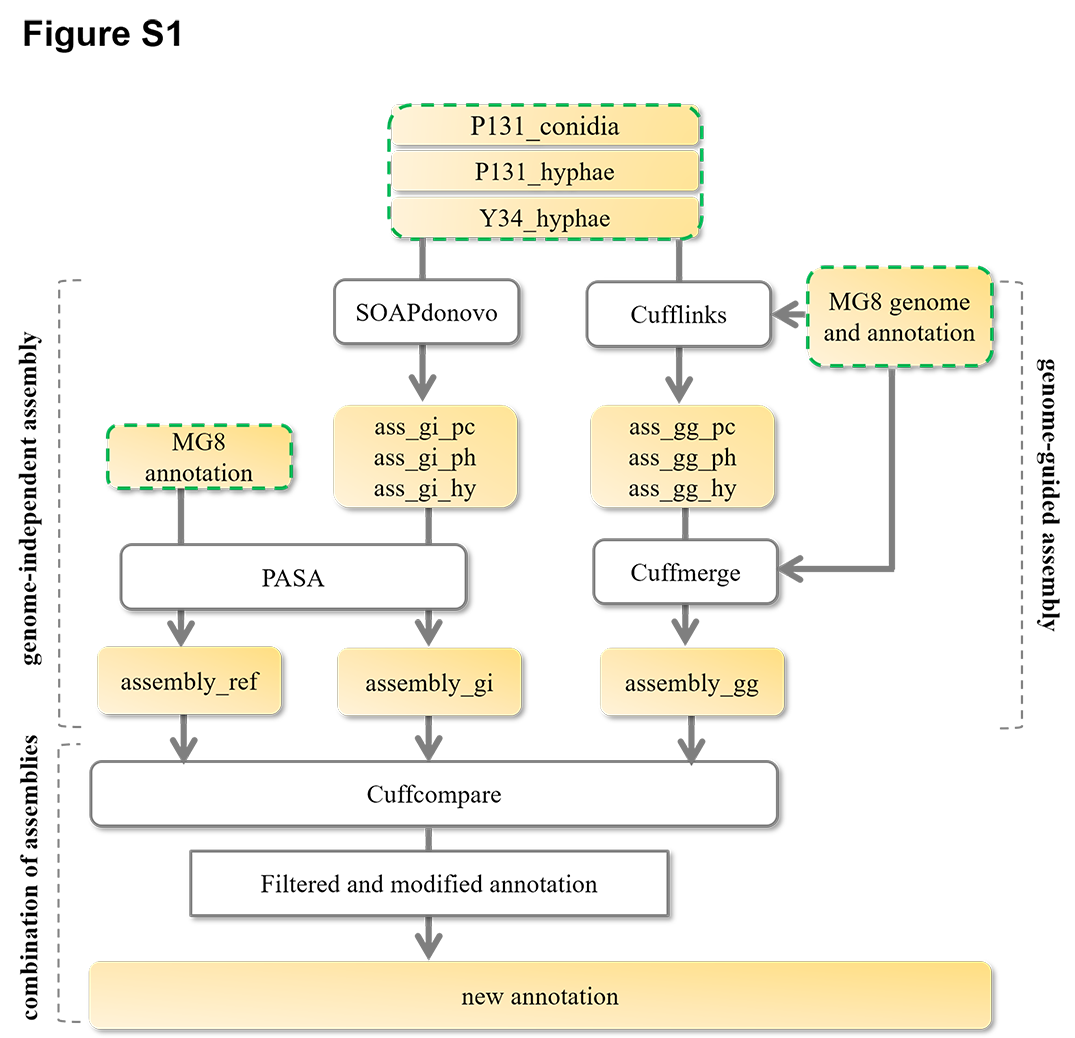

Supplement: Supplementary Figure 1 — Pipeline showing assembly strategy. A hybrid strategy is employed to assemble RNA-Seq data from three samples. This strategy includes three steps: genome-independent assembly, genome-guided assembly and a combination of assemblies. Boxes with brown backgrounds indicate data sets, boxes with white background mean processing procedures, and boxes with broken green frames are original data used in the assemblies. [file Image_1.TIF]

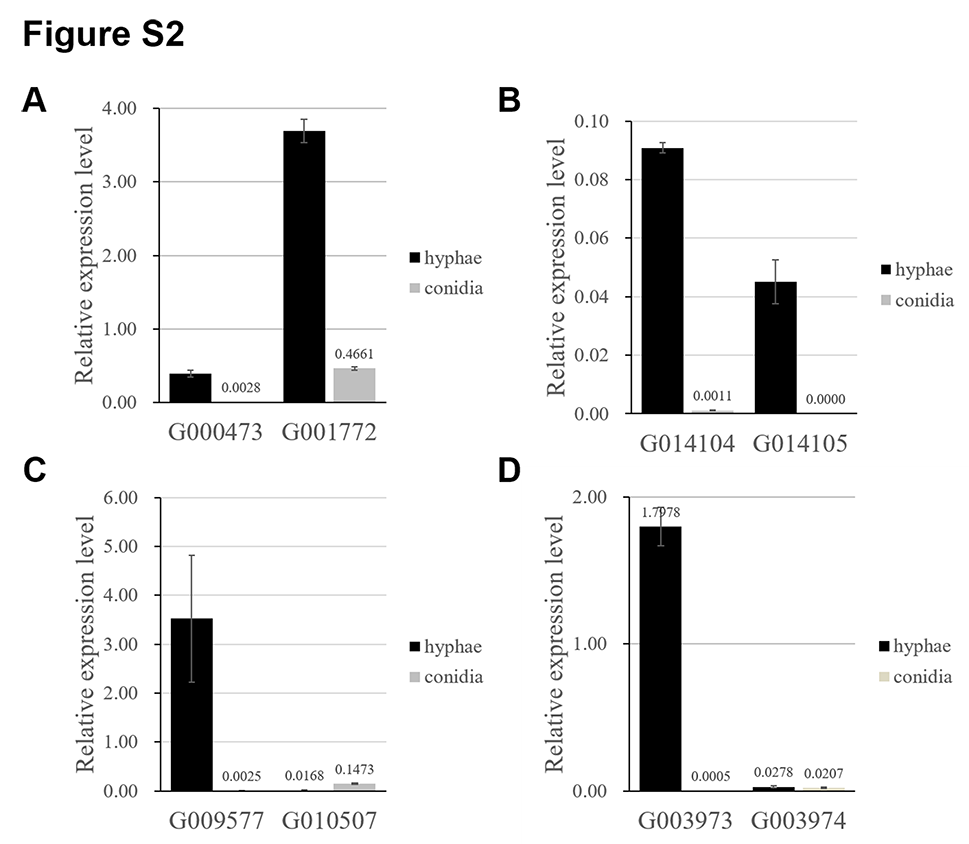

Supplement: Supplementary Figure 2 — Relative expression levels of different transcripts. Bar graphs showed the relative expressions of (A) G000473 and G001772 (MGG_16180), (B) G014104 and G014105 (MGG_04005), (C) G009577 and G010507 (MGG_06585), and (D) G003973 and G003974 (MGG_15773) in mycelium and conidia with quantitative RT-PCR assays, respectively. [file Image_2.TIF]

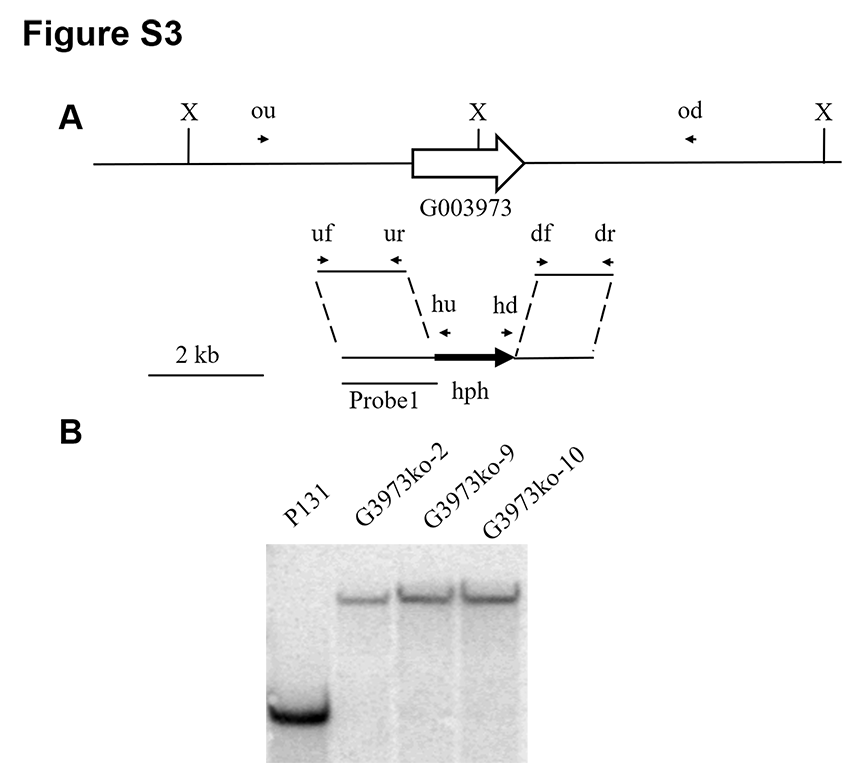

Supplement: Supplementary Figure 3 — Gene knockout of G003973 lncRNA and validation. (A) G003973 and its gene replacement vector (constructed by replacing G003973 with hph). The position of primers ou, od, uf, ur, df, dr, hu, and hd are indicated with small arrows. X, XhoI. (B) DNA gel blot on XhoI-digested DNAs of strains P131 and the G003973 deletion mutants G3973-2, G3973-9, and G3973-10 hybridizing against probe 1 in panel (A). The estimated sizes of each band are marked at right. [file Image_3.TIF]

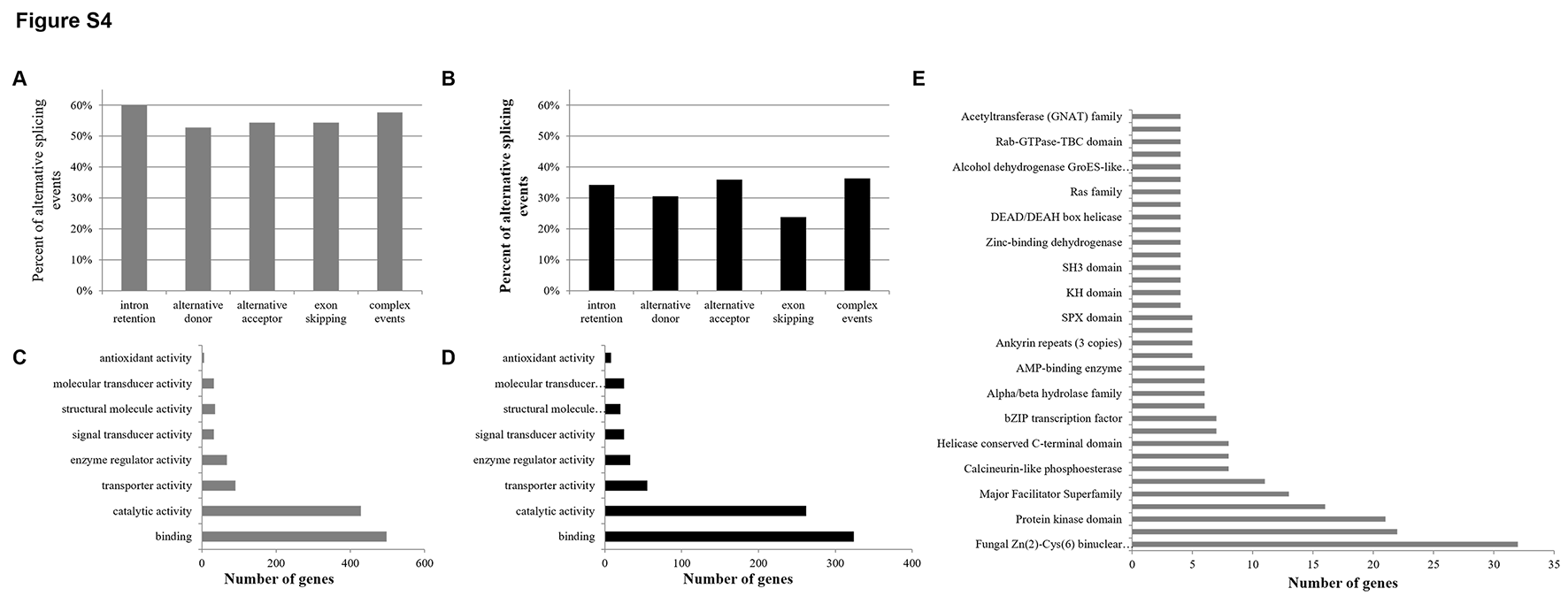

Supplement: Supplementary Figure 4 — Comparison of inter-tissue and inter-isolate alternative splicing variations. (A,B) The extent of alternative splicing events that are significantly regulated between tissues and isolates. (C,D) GO categories of genes with significantly regulated alternative splicing events between tissues and isolates. (E) Pfam domains of genes with significantly regulated alternative splicing events between tissues. [file Image_4.TIF]

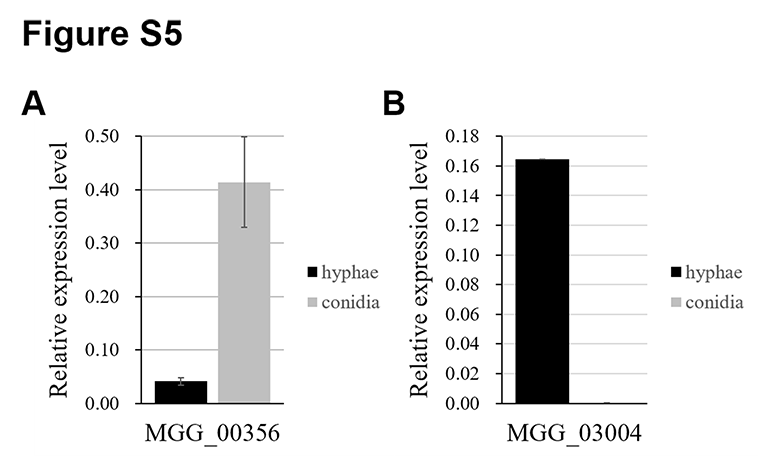

Supplement: Supplementary Figure 5 — Relative expression levels of different genes. Bar graphs showed the relative expression levels of (A) MGG_00356 and (B) MGG_03004 in hyphae and conidia, respectively. [file Image_5.TIF]

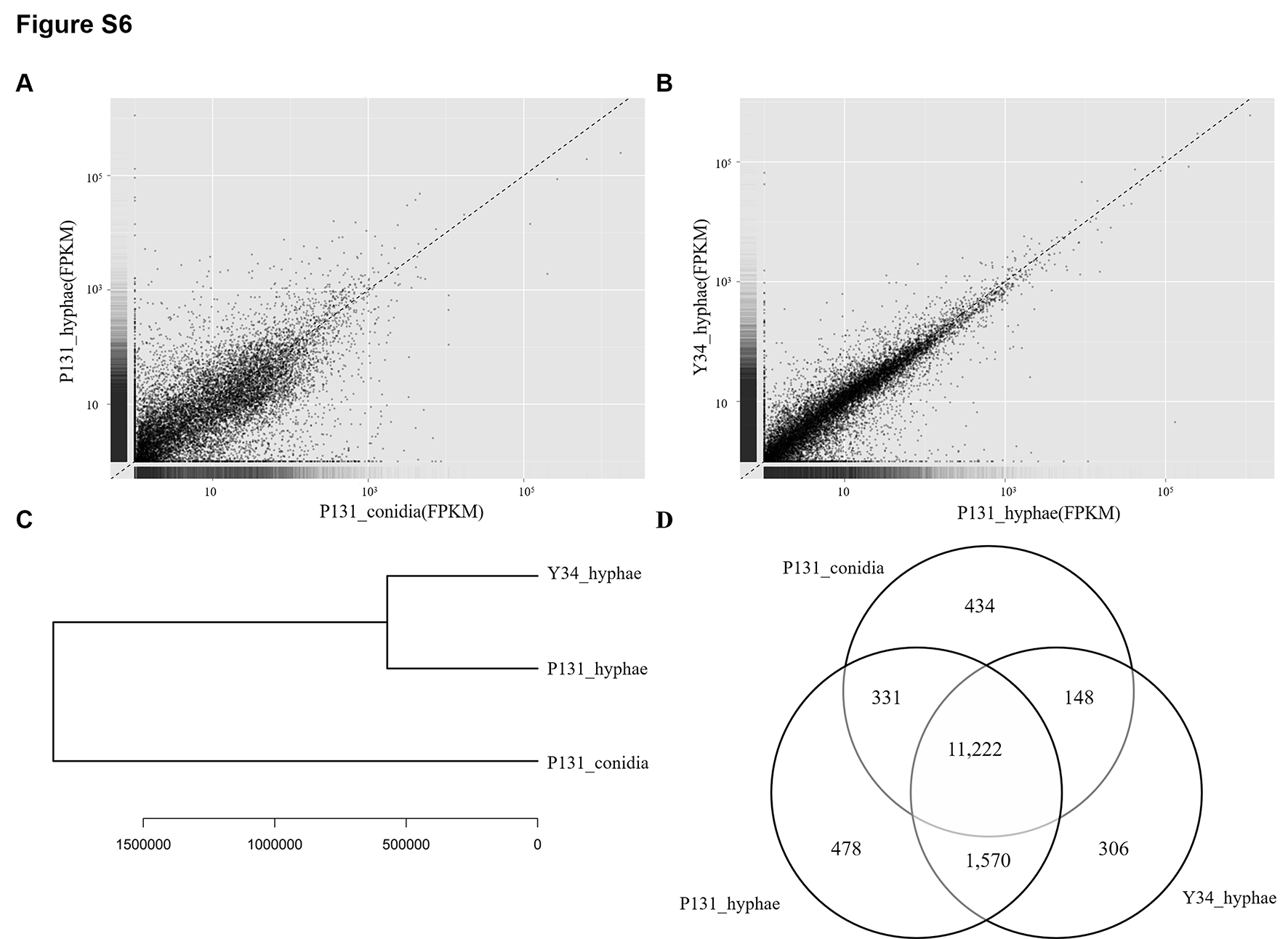

Supplement: Supplementary Figure 6 — Overall comparisons of gene abundances. (A,B) are pair-wise scatterplots of gene expressions. The scatterplot indicates the global change of gene abundances between the two samples. Gene abundances are represented by FPKM. (C) Overall similarities between the samples are indicated by the clustering based on Euclidean distances. (D) Venn diagram indicating expressed genes in the three samples. Number in each set means gene number. [file Image_6.TIF]

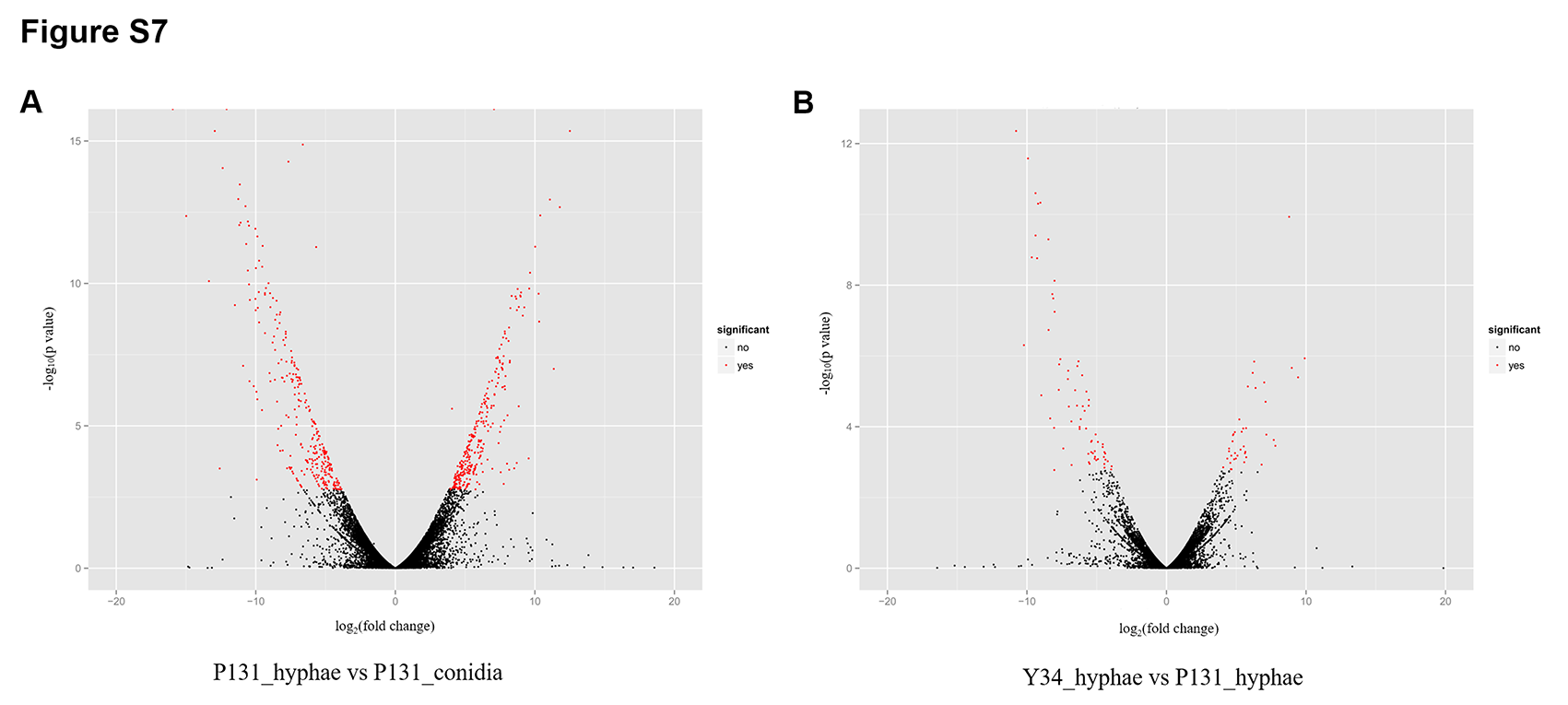

Supplement: Supplementary Figure 7 — Volcano plots depicting the relationship between fold-change and significance. Each point represents a gene in our new annotation. Red points indicate that their expression levels are significantly different between samples. (A) Comparison between P131_hyphae and P131_conidia. (B) Comparison between Y34_hyphae and P131_hyphae. [file Image_7.TIF]

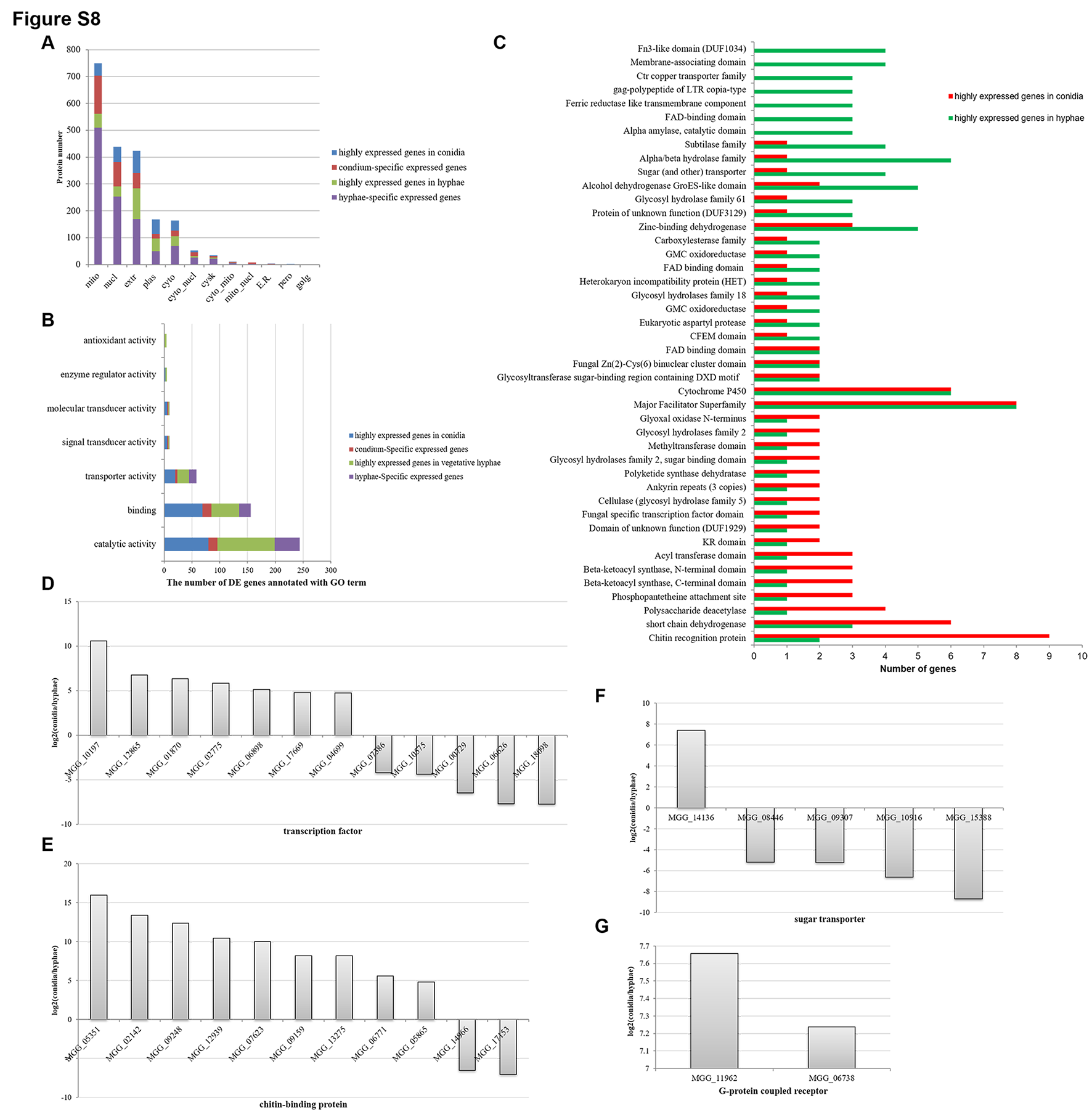

Supplement: Supplementary Figure 8 — Differentially expressed (DE) genes between P131 conidia and P131 hyphae. (A) Bar graph depicts subcellular localizations of proteins encoded by DE genes. (B) GO categories of DE gene-encoded proteins. (C) Pfam categories of highly expressed genes in conidia or hyphae. (D–G) Log2 (fold change) of highly expressed genes annotated as (D) transcription factors, (E) chitin-binding proteins, (F) sugar transporters, and (G) G-protein coupled receptors. [file Image_8.TIF]

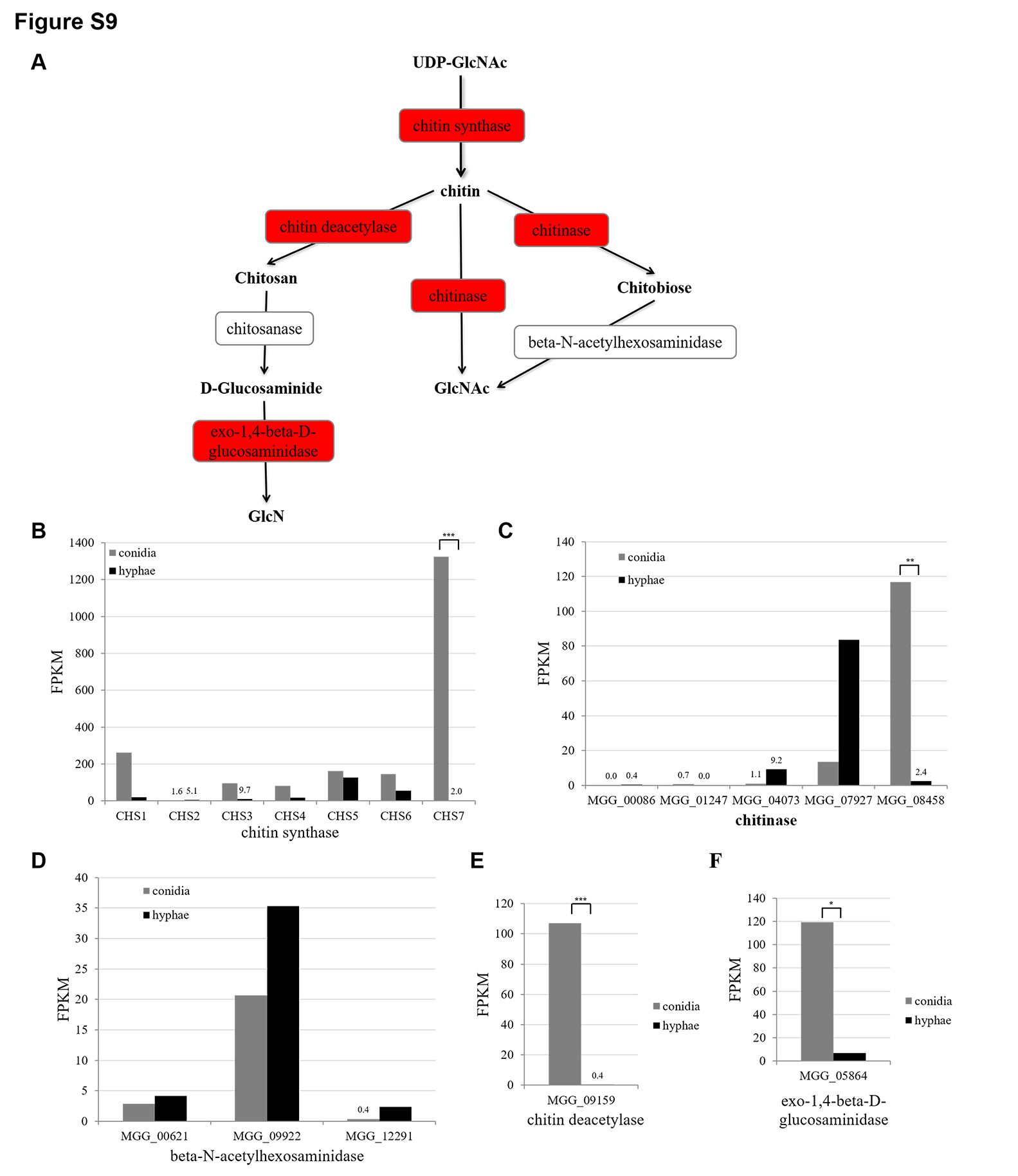

Supplement: Supplementary Figure 9 — Regulations of chitin synthesis and degradation. (A) Pathways of chitin synthesis and degradation. Genes represented by red boxes are up-regulated in conidia compared with hyphae. (B–F) Comparison of gene expression levels of CHS, chitinases, beta-N-acetyl hexosaminidases, chitin deacetylase and exo-1,4-beta-D-glucosaminidase in conidia and hyphae. [file Image_9.TIF]
