# Supplementary material for: Transcriptional Landscapes of Long Non-coding RNAs and Alternative Splicing in Pyricularia oryzae Revealed by RNA-Seq
Source: Front Plant Sci. 2021 Sep 8;12:723636. doi: 10.3389/fpls.2021.723636 (PMC8475275; doi:10.3389/fpls.2021.723636)
Supplement: Supplementary Table 1 — Summary of RNA-Seq data. [file Table_1.DOCX]

**Table S1 Summary of RNA-Seq data.**

|  | Number of reads | Read length(bp) | Paired-end insert size(bp) |
| --- | --- | --- | --- |
| P131_conidia | 13,133,334 | 90 | 200 |
| P131_hyphae | 48,125,694 | 75 | 200 |
| Y34_hyphae | 47,846,570 | 75 | 200 |
