# Supplementary material for: Transcriptional Landscapes of Long Non-coding RNAs and Alternative Splicing in Pyricularia oryzae Revealed by RNA-Seq
Source: Front Plant Sci. 2021 Sep 8;12:723636. doi: 10.3389/fpls.2021.723636 (PMC8475275; doi:10.3389/fpls.2021.723636)
Supplement: Supplementary Table 2 — Summary of assemblies. [file Table_2.DOCX]

**Table S2 Summary of assemblies.**

|  | MG8 | assembly_gi^a^ | assembly_ref^b^ | assembly_gg^c^ | new annotation |
| --- | --- | --- | --- | --- | --- |
| **Genes** |  |  |  |  |  |
| Number of genes | 12,827 | 19,820 | 12,822 | 13,677 | 16,192 |
| Number of genes + strand | 6,423 | 3,779 | 6,421 | 6,196 | 7,179 |
| Number of genes - strand | 6,404 | 3,841 | 6,401 | 6,190 | 7,209 |
| Number of genes no strand | 0 | 12,200 | 0 | 1,291 | 1,804 |
| Mean gene length(bp) | 2,011 | 1,263 | 2,159 | 2,383 | 2,046 |
| Number of genes with multiple isoforms | 155 | 970 | 686 | 3,527 | 2,358 |
| Number of genes with multiple exons | 10,332 | 7,620 | 10,416 | 10,164 | 11,513 |
|  |  |  |  |  |  |
| **Transcripts** |  |  |  |  |  |
| Number of transcripts | 12,991 | 20,903 | 13,583 | 19,080 | 19,418 |
| Mean transcript length(bp) | 1,802 | 1,258 | 1,997 | 2,143 | 1,928 |
| Number of multiple exon transcripts | 10,481 | 8,703 | 11,148 | 14,507 | 14,374 |

a: assembly result by genome-independent method.

b: The MG8 annotated updated by PASA.

c: assembly result by genome-guided method.
