# Supplementary material for: Transcriptional Landscapes of Long Non-coding RNAs and Alternative Splicing in Pyricularia oryzae Revealed by RNA-Seq
Source: Front Plant Sci. 2021 Sep 8;12:723636. doi: 10.3389/fpls.2021.723636 (PMC8475275; doi:10.3389/fpls.2021.723636)
Supplement: Supplementary file 15 [file Data_Sheet_1.DOCX]

**Supplementary Results**

**RNA-seq assembly**

To improve the genome annotation of *M. oryzae*, we reconstructed a new transcriptome with a hybrid assembly strategy that merges genome-guided and genome-independent methods as follows (Figure S1).

*Genome-independent assembly*

SOAPdenovo (Li et al., 2010) was employed to assemble each RNA-Seq data independently. 26,866, 24,557 and 22,447 scaffolds were generated for P131_conidia, P131_hyphae and Y34_hyphae, respectively. The corresponding assembly sizes were 15, 18 and 19 Mb. N50 values were 816, 1,135 and 1,433 bp, respectively. The assembled scaffolds of the three samples were merged by PASA (an acronym for Program to Assemble Spliced Alignments) (Haas et al., 2003). Scaffolds of P131_conidia, P131_hyphae and Y34_hyphae were aligned to chromosomes of *M. oryzae* 70-15 by GMAP (Wu and Watanabe, 2005) and clustered by PASA (“assembly_gi” in Figure S1, and Table S2). A total of 20,903 assembled sequences were created by PASA and grouped into 19,820 clusters. Assembled sequences in the same cluster may be alternatively spliced isoforms.

We incorporated the PASA assembled sequences into protein-coding genes predicted in MG8 annotation and yielded an updated annotation. Processes of correcting exon boundaries, adding UTRs and modifying alternatively spliced isoforms were also implemented by PASA. The updated annotation (“assembly_ref” in Figure S1) produced 13,583 transcripts derived from 12,822 genes. Six hundred eighty-six genes were found with multiple forms of transcripts. The average length of transcripts also increased from 1,802 bp to 1,997 bp (Table S2).

*Genome-guided assembly*

Prior to genome-guided assembly, RNA-Seq raw reads from P131_conidia, P131_hyphae and Y34_hyphae were mapped separately onto seven chromosomes and the unplaced genomic scaffold of the strain 70-15 (i.e. Chromosome_8.8 in MG8 annotation) by Tophat (Trapnell et al., 2009). Unlike mapping RNA-Seq short reads against a reference transcriptome (e.g. cDNA.), a key challenge for mapping against a genome is that many reads span exon-exon junctions. As a result, there may be large gaps between an intron-spanning read and the reference genome. To overcome this difficulty, Tophat first mapped non-junction reads using an ultra-fast short read mapping program Bowtie2 (Langmead et al., 2009). After that, Tophat aligned unmapped reads to a database of possible junctions to confirm intron-spanning reads. The percentages of mapped reads of P131_conidia, P131_hyphae and Y34_hyphae were 94.6%, 92.7% and 92.0%, respectively. These alignments were used for the subsequent genome-guided assembly.

To construct the transcriptome of *M. oryzae*, Cufflinks (Trapnell et al., 2010) that was designed to maximize precision (Garber et al., 2011), was employed to assemble the mapped reads. Since some genes are expressed at low levels, they might be only partially covered in RNA-Seq data. To compensate for the low coverage, we performed Reference Annotation Based Transcript (RABT) assembly (Roberts et al., 2011). In the RABT assembly, transcripts in MG8 annotation were used to generate faux-read alignments. Some features missed in sequencing data due to low coverage could be captured by faux-reads, which could be a great aid to assembly. Read alignments of P131_conidia, P131_hyphae and Y34_hyphae created by Tophat together with faux-read alignments were provided to Cufflinks to generate transcripts, respectively. Assemblies of these three data were then merged together with Cuffmerge. After merging, a total of 19,080 transcripts (derived from 13,677 genes) were generated (i.e. “assembly_gg” in Figure S1, and Table S2).

*Combination of the assemblies*

The prior assemblies (i.e. “assembly_gi”, “assembly_gg” and “assembly_ref” in Figure S1) were filtered before combination. Strand information of single-exon transcripts was removed. Transcripts spanning more than two genes (in MG8 annotation) were discarded. Multiple exon-containing genes with unusual splice sites (i.e. splice sites except for GT/AG, GC/AC and AT/AC) at intron boundaries were filtered as well.

Then the program Cuffcompare was employed to combine assemblies. Based on the comparison with “assembly_ref” (the reference annotation), combined transcripts can be categorized into different match types, which were described by class code in Cuffcompare. For example, class code “j” indicates novel isoforms sharing at least one splice junction with a known transcript. We extracted transcripts with complete matches of all introns (class code “=”) and inter-genic transcripts (class code “p” or “u”). We also kept multiple exon transcripts that fallen entirely within reference introns (class code “i”) or overlapped with reference genes (class code “o” or “x”). If a single exon transcript overlapped with a reference gene and this reference gene contained only a single exon (class code “o”), we extracted as well.

Novel isoforms (class code “j”) were also extracted. As there may be lots of reads originating from incompletely spliced transcripts in RNA-Seq data, these reads tend to confound assembly. For the genome-guided assembly method, Cufflinks can reduce the influence of these misleading reads using a parameter “--pre-mrna-fraction”. However, for the genome-independent assembly method, it was difficult to reduce such influence. To avoid this issue, novel isoforms from the genome-independent assembly were discarded if they did not contain new putative exon-exon junctions.

We then combined these extracted transcripts as a new set of genes. Finally, the ultimate annotated transcriptome of *M. oryzae* was constructed (“new annotation” in Figure S1).

**Gene quantification analysis**

To quantify gene expression, we firstly re-mapped RNA-Seq reads of P131_conidia, P131_hyphae and Y34_hyphae to the genome of 70-15 with Tophat (Trapnell et al., 2009). Our new annotation was used to improve the effect of read mapping. Tophat would align reads to our newly constructed transcriptome first. Reads that could not be fully mapped to the transcriptome were then mapped to the genome sequence. After this round of mapping, 170,264, 731,231 and 628,584 reads, which could not mapped in the previous step, were rescued for P131_conidia, P131_hyphae and Y34_hyphae, respectively. Cuffdiff (Trapnell et al., 2010) was employed to estimate gene abundances and then identify significant changes in gene expression. Gene abundances were measured with fragments per kilobase of exon per million fragments mapped (FPKM) (Trapnell et al., 2010) (Figure S6). To evaluate the overall similarities among these three samples, we calculated pairwise Euclidean distances using gene abundance values. These three samples were further clustered based on Euclidean distances. The result showed that the global pattern of gene expression in hyphae was highly distinctive from that in conidia (Figure S6).

*Genes induced in conidia*

We compared the gene expression levels between P131_conidia and P131_hyphae. Of 16,192 genes, 664 genes were induced in conidia, including 291 genes and 373 genes that were significantly highly expressed and were specifically expressed (>1 FPKM) in conidia, respectively. Among the induced genes, 189 genes encode mitochondrial proteins, which occupied 29%. Additionally, 148, 139 and 71 genes encoded nuclear, extracellular proteins and plasma membrane proteins, respectively.

To understand molecular functions of the genes highly expressed in conidia, we performed GO enrichment analysis and Pfam domain identification, which were accepted as a *de facto* standard for gene functional description (Figure S8 B, C, and Table S5). A total of 96 genes were annotated to have catalytic activity, including 38 hydrolases, 40 oxidoreductases, 24 transferases and 8 lyases. Eighty-five genes were annotated to have binding activity, including binding proteins for ion (22 genes), cofactor (17 genes), nucleotide (15 genes), tetrapyrrole (8 genes), carbohydrate (8 genes), etc. 24 genes had transporter activity. Notably, two genes encoding G-protein coupled receptors (GPCR) were significantly induced in conidia (MGG_11962 and MGG_06738) (Figure S8 G). 15 transcription factors were induced in conidia (Park et al., 2008). The above-mentioned data suggested that the genes induced in conidia play important roles in sensing and transmitting environmental stimuli to regulate gene expression for conidiation and also possibly for pathogenesis.

Several genes that were previously reported important for conidial development were significantly highly expressed in conidia, including *ACR1* (MGG_09847), *MoFLP1* (MGG_02884) and *MoCON6* (MGG_02246). *ACR1* encodes a glutamine-rich domain protein that is important for conidiogenesis, and its deletion can result in the formation of head-to-tail conidia (Lau and Hamer, 1998). *MoFLP1* encodes a fasciclin-like protein that is important for conidiation (Liu et al., 2009). *MoCON6*, whose ortholog in *N. crassa* plays a unique role in conidiation, is not expressed in mycelium (Madi et al., 1994).

Some genes involved in pathogenicity were significantly highly expressed in conidia, including *MoSFL1* (MGG_06971), *ICL1* (MGG_04895) and *GAS1* (MGG_12337). *MoSFL1* is a gene important for virulence (Li et al., 2011). *ICL1*, encoding isocitrate lyase, is also required for full virulence (Wang et al., 2003). The *GAS*1 gene, important for appressorial penetration and lesion development,was reported to be highly expressed in appressorium (Xue et al., 2002), could also be detected in conidia and vegetative hyphae. Some conidium-specific expressed genes were also important for pathogenicity as well, including *SLP1* (MGG_10097), *PTH11* (MGG_05871) and *Erl1* (MGG_02549). *SLP1* encodes a glycosylated effector protein that functions at the plant-fungal interface to suppresses chitin-triggered immunity (Mentlak et al., 2012, Chen et al., 2014). PTH11 is a transmembrane protein involved in host surface recognition (DeZwaan et al., 1999). Erl1 is required for full root virulence (Heupel et al., 2010).

*M. oryzae* genome contains seven genes for chitin synthases (*CHS*) for the synthesis of chitin that is a major component in fungal cell walls (Lenardon et al., 2010, Kong et al., 2012). Figure 5B shows expression abundances of the seven CHS genes in conidia and hyphae, which are highly similar to the expression profiles assayed by qRT-PCR previously (Kong et al., 2012), supporting the validity of our data. *CHS7* (MGG_06064) is a gene important for appressorial penetration and invasive growth of infection hyphae (Kong et al., 2012). The gene is highly expressed in conidia but lowly in hyphae. It may be regulated by transcription factor Con7p (Odenbach et al., 2007), and the expression of *CHS7* is consistent with *con7* (MGG_05287, 42-fold change of FPKM). In addition to chitin synthases, several genes involved in the degradation of chitin are up-regulated in conidia as well (Figure 5), including chitin deacetylase (MGG_09159), chitinase (MGG_08458) and exo-1,4-beta-D-glucosaminidase (MGG_05864). Interestingly, MGG_08458 was highly expressed in conidia, whereas another chitinase MGG_07927 were highly expressed in hyphae.

*CBP1* (MGG_12939) and *CBP2* (MGG_07623) are two chitin-binding protein-encoding genes. CBP1 is an extracellular protein and plays an important role in hydrophobic surface recognition during appressorium differentiation (Kamakura et al., 2002). CBP2 is located in the cell wall and phenotypes of the *∆cbp2* mutant are not affected (Breth et al., 2013). Notably, both of CBP1 and CBP2 are extremely induced in conidia as compared to hyphae (1374 and 1017-fold change of FPKM, respectively). The two genes may be regulated by several transcription factors, such as Con7p (Odenbach et al., 2007) and Tra1p (Breth et al., 2013). *TRA1* (MGG_10197) was also highly expressed in conidia (1560-fold change of FPKM). CBP2 is also regulated by a homeobox transcription factor encoded by *MoHOX2* (Kim et al., 2009, Kim and Lee, 2012). In addition, seven putative CBP family genes (MGG_13275, MGG_05351, MGG_05865, MGG_09248, MGG_09159, MGG_06771 and MGG_02142) were also induced in conidia.

*Gene induced in vegetative hyphae*

A total of 1,444 genes were identified to be induced in vegetative hyphae, including 302 highly expressed genes and 1,142 hyphae-specific expressed genes (>1 FPKM). 561, 291, 284 and 97 genes were predicted to encode mitochondrial, nuclear and extracellular and plasma membrane proteins, respectively. One hundred forty-eight genes were annotated to have catalytic activity, including 69 hydrolases, 59 oxidoreductases, 23 transferases and 8 lyases. 71 genes were annotated with binding activity, including binding proteins for ion (31 genes), cofactor (13 genes), nucleotide (11 genes), tetrapyrrole (11 genes), carbohydrate (7 genes). Thirty-four genes were with transporter activity. Additionally, 26 transcription factors were identified to be induced in hyphae (Park et al., 2008).

Some highly expressed genes were involved in pathogenicity, e.g. *PTH3* (MGG_07528) (Sweigard et al., 1998), *MgSM1* (MGG_05344) (Jeong et al., 2007) and *OMO1* (MGG_04212) (Hof et al., 2007). *MgSM1* encodes an extracellular protein, which is required for virulence and down-regulated during appressorium formation (Oh et al., 2008). Some genes involved in pathogenicity are explicitly expressed in hyphae. *Avr-Pizt* (MGG_18041) (Li et al., 2009) and *Avr-Pita* (MGG_15370) (Jia et al., 2000) were two avirulence genes, which could be detected specifically in P131_hyphae. However, both were expressed at low levels (*Avr-Pizt*, 1 FPKM; *Avr-Pita*, 0.09 FPKM). Mutant of MGG_12026 (with 4.2 PFKM in P131_hyphae) affected appressorium formation and pathogenicity (Jeon et al., 2007).

*DE genes between isolates*

Gene expression levels were also compared between P131_hyphae and Y34_hyphae. There were 454 and 809 genes that were uniquely expressed in Y34_hyphae and P131_hyphae, respectively. There were 40 genes highly expressed in Y34 and 67 genes highly expressed in P131, respectively. Among them are *MPG1* (MGG_10315) and MGG_10510. *MPG1* encodes a fungal hydrophobin involved in surface recognition (Talbot et al., 1993, Beckerman and Ebbole, 1996), suggesting these two strains might respond differently to environmental clues. The yeast ortholog of MGG_10510 is Rny1p, which is required for tRNA cleavage (Nunes et al., 2011). tRNA cleavage around the anticodon loop results in a class of small RNAs (tRNA-derived RNA fragments), which is considered a novel mechanism for regulation of protein synthesis (Jochl et al., 2008). tRNA cleavage occurs in response to specific stresses, such as oxidative stress and nutrient starvation (Jochl et al., 2008, Thompson et al., 2008). In addition, tRNA cleavage is induced during conidiation in *A. fumigatus*. Conidiation, appressorium formation and pathogenicity of *M. oryzae* are all affected in the mutant of MGG_10510 (Jeon et al., 2007). Two putative transcription factor-encoding genes are also identified among these gene, i.e. MGG_05501, and MGG_09841 (Park et al., 2008). As reported previously (Xue et al., 2012), Y34 and P131 are different in conidiation, vegetative growth, virulence and pathotypes. These isolated-specific or differentially expressed genes may contribute to the phenotypic changes between the two field isolates and to their environmental adaptation.

***Alternative splicing regulation between isolates***

The variation of alternative splicing between P131 and Y34 was assessed as well. A similar method as used in the comparison between conidia and hyphae was employed here. A total of 987 alternative splicing events were regulated. Between ~24% and 36% of alternative splicing events showed significant inter-isolate variation, depended on the event pattern (Figure S4 B, D). These frequencies were about two-fold less than the 52-60% of events showed that variation between conidia and hyphae. Compared with inter-tissue comparison, the percentage of intron retention events showed inter-isolate variation was the highest as well. However, the alternative splicing pattern showing the lowest percentage variation was exon skipping.

**References**

Beckerman, J. L. and D. J. Ebbole (1996). "MPG1, a gene encoding a fungal hydrophobin of Magnaporthe grisea, is involved in surface recognition." Mol Plant Microbe Interact **9**(6): 450-456.

Breth, B., D. Odenbach, A. Yemelin, N. Schlinck, M. Schroder, M. Bode, L. Antelo, K. Andresen, E. Thines and A. J. Foster (2013). "The role of the Tra1p transcription factor of Magnaporthe oryzae in spore adhesion and pathogenic development." Fungal Genet Biol.

Chen, X. L., T. Shi, J. Yang, W. Shi, X. Gao, D. Chen, X. Xu, J. R. Xu, N. J. Talbot and Y. L. Peng (2014). "N-Glycosylation of Effector Proteins by an alpha-1,3-Mannosyltransferase Is Required for the Rice Blast Fungus to Evade Host Innate Immunity." Plant Cell **26**(3): 1360-1376.

DeZwaan, T. M., A. M. Carroll, B. Valent and J. A. Sweigard (1999). "Magnaporthe grisea pth11p is a novel plasma membrane protein that mediates appressorium differentiation in response to inductive substrate cues." Plant Cell **11**(10): 2013-2030.

Garber, M., M. G. Grabherr, M. Guttman and C. Trapnell (2011). "Computational methods for transcriptome annotation and quantification using RNA-seq." Nat Methods **8**(6): 469-477.

Haas, B. J., A. L. Delcher, S. M. Mount, J. R. Wortman, R. K. Smith, Jr., L. I. Hannick, R. Maiti, C. M. Ronning, D. B. Rusch, C. D. Town, S. L. Salzberg and O. White (2003). "Improving the Arabidopsis genome annotation using maximal transcript alignment assemblies." Nucleic Acids Res **31**(19): 5654-5666.

Heupel, S., B. Roser, H. Kuhn, M. H. Lebrun, F. Villalba and N. Requena (2010). "Erl1, a novel era-like GTPase from Magnaporthe oryzae, is required for full root virulence and is conserved in the mutualistic symbiont Glomus intraradices." Mol Plant Microbe Interact **23**(1): 67-81.

Hof, C., K. Eisfeld, K. Welzel, L. Antelo, A. J. Foster and H. Anke (2007). "Ferricrocin synthesis in Magnaporthe grisea and its role in pathogenicity in rice." Mol Plant Pathol **8**(2): 163-172.

Jeon, J., S. Y. Park, M. H. Chi, J. Choi, J. Park, H. S. Rho, S. Kim, J. Goh, S. Yoo, J. Y. Park, M. Yi, S. Yang, M. J. Kwon, S. S. Han, B. R. Kim, C. H. Khang, B. Park, S. E. Lim, K. Jung, S. Kong, M. Karunakaran, H. S. Oh, H. Kim, S. Kang, W. B. Choi and Y. H. Lee (2007). "Genome-wide functional analysis of pathogenicity genes in the rice blast fungus." Nat Genet **39**(4): 561-565.

Jeong, J. S., T. K. Mitchell and R. A. Dean (2007). "The Magnaporthe grisea snodprot1 homolog, MSP1, is required for virulence." FEMS Microbiol Lett **273**(2): 157-165.

Jia, Y., S. A. McAdams, G. T. Bryan, H. P. Hershey and B. Valent (2000). "Direct interaction of resistance gene and avirulence gene products confers rice blast resistance." EMBO J **19**(15): 4004-4014.

Jochl, C., M. Rederstorff, J. Hertel, P. F. Stadler, I. L. Hofacker, M. Schrettl, H. Haas and A. Huttenhofer (2008). "Small ncRNA transcriptome analysis from Aspergillus fumigatus suggests a novel mechanism for regulation of protein synthesis." Nucleic Acids Res **36**(8): 2677-2689.

Kamakura, T., S. Yamaguchi, K. Saitoh, T. Teraoka and I. Yamaguchi (2002). "A novel gene, CBP1, encoding a putative extracellular chitin-binding protein, may play an important role in the hydrophobic surface sensing of Magnaporthe grisea during appressorium differentiation." Mol Plant Microbe Interact **15**(5): 437-444.

Kim, K. S. and Y. H. Lee (2012). "Gene Expression Profiling during Conidiation in the Rice Blast Pathogen Magnaporthe oryzae." PLoS One **7**(8): e43202.

Kim, S., S. Y. Park, K. S. Kim, H. S. Rho, M. H. Chi, J. Choi, J. Park, S. Kong, J. Goh and Y. H. Lee (2009). "Homeobox transcription factors are required for conidiation and appressorium development in the rice blast fungus Magnaporthe oryzae." PLoS Genet **5**(12): e1000757.

Kong, L. A., J. Yang, G. T. Li, L. L. Qi, Y. J. Zhang, C. F. Wang, W. S. Zhao, J. R. Xu and Y. L. Peng (2012). "Different chitin synthase genes are required for various developmental and plant infection processes in the rice blast fungus Magnaporthe oryzae." PLoS Pathog **8**(2): e1002526.

Langmead, B., C. Trapnell, M. Pop and S. L. Salzberg (2009). "Ultrafast and memory-efficient alignment of short DNA sequences to the human genome." Genome Biol **10**(3): R25.

Lau, G. W. and J. E. Hamer (1998). "Acropetal: a genetic locus required for conidiophore architecture and pathogenicity in the rice blast fungus." Fungal Genet Biol **24**(1-2): 228-239.

Lenardon, M. D., C. A. Munro and N. A. Gow (2010). "Chitin synthesis and fungal pathogenesis." Curr Opin Microbiol **13**(4): 416-423.

Li, G., X. Zhou, L. Kong, Y. Wang, H. Zhang, H. Zhu, T. K. Mitchell, R. A. Dean and J. R. Xu (2011). "MoSfl1 is important for virulence and heat tolerance in Magnaporthe oryzae." PLoS One **6**(5): e19951.

Li, R., H. Zhu, J. Ruan, W. Qian, X. Fang, Z. Shi, Y. Li, S. Li, G. Shan, K. Kristiansen, H. Yang and J. Wang (2010). "De novo assembly of human genomes with massively parallel short read sequencing." Genome Res **20**(2): 265-272.

Li, W., B. Wang, J. Wu, G. Lu, Y. Hu, X. Zhang, Z. Zhang, Q. Zhao, Q. Feng, H. Zhang, Z. Wang, G. Wang, B. Han and B. Zhou (2009). "The Magnaporthe oryzae avirulence gene AvrPiz-t encodes a predicted secreted protein that triggers the immunity in rice mediated by the blast resistance gene Piz-t." Mol Plant Microbe Interact **22**(4): 411-420.

Liu, T. B., G. Q. Chen, H. Min and F. C. Lin (2009). "MoFLP1, encoding a novel fungal fasciclin-like protein, is involved in conidiation and pathogenicity in Magnaporthe oryzae." J Zhejiang Univ Sci B **10**(6): 434-444.

Madi, L., D. J. Ebbole, B. T. White and C. Yanofsky (1994). "Mutants of Neurospora crassa that alter gene expression and conidia development." Proc Natl Acad Sci U S A **91**(13): 6226-6230.

Mentlak, T. A., A. Kombrink, T. Shinya, L. S. Ryder, I. Otomo, H. Saitoh, R. Terauchi, Y. Nishizawa, N. Shibuya, B. P. Thomma and N. J. Talbot (2012). "Effector-mediated suppression of chitin-triggered immunity by magnaporthe oryzae is necessary for rice blast disease." Plant Cell **24**(1): 322-335.

Nunes, C. C., M. Gowda, J. Sailsbery, M. Xue, F. Chen, D. E. Brown, Y. Oh, T. K. Mitchell and R. A. Dean (2011). "Diverse and tissue-enriched small RNAs in the plant pathogenic fungus, Magnaporthe oryzae." BMC Genomics **12**: 288.

Odenbach, D., B. Breth, E. Thines, R. W. Weber, H. Anke and A. J. Foster (2007). "The transcription factor Con7p is a central regulator of infection-related morphogenesis in the rice blast fungus Magnaporthe grisea." Mol Microbiol **64**(2): 293-307.

Oh, Y., N. Donofrio, H. Pan, S. Coughlan, D. E. Brown, S. Meng, T. Mitchell and R. A. Dean (2008). "Transcriptome analysis reveals new insight into appressorium formation and function in the rice blast fungus Magnaporthe oryzae." Genome Biol **9**(5): R85.

Park, J., S. Jang, S. Kim, S. Kong, J. Choi, K. Ahn, J. Kim, S. Lee, B. Park, K. Jung, S. Kang and Y. H. Lee (2008). "FTFD: an informatics pipeline supporting phylogenomic analysis of fungal transcription factors." Bioinformatics **24**(7): 1024-1025.

Roberts, A., H. Pimentel, C. Trapnell and L. Pachter (2011). "Identification of novel transcripts in annotated genomes using RNA-Seq." Bioinformatics **27**(17): 2325-2329.

Sweigard, J. A., A. M. Carroll, L. Farrall, F. G. Chumley and B. Valent (1998). "Magnaporthe grisea pathogenicity genes obtained through insertional mutagenesis." Mol Plant Microbe Interact **11**(5): 404-412.

Talbot, N. J., D. J. Ebbole and J. E. Hamer (1993). "Identification and characterization of MPG1, a gene involved in pathogenicity from the rice blast fungus Magnaporthe grisea." Plant Cell **5**(11): 1575-1590.

Thompson, D. M., C. Lu, P. J. Green and R. Parker (2008). "tRNA cleavage is a conserved response to oxidative stress in eukaryotes." RNA **14**(10): 2095-2103.

Trapnell, C., L. Pachter and S. L. Salzberg (2009). "TopHat: discovering splice junctions with RNA-Seq." Bioinformatics **25**(9): 1105-1111.

Trapnell, C., B. A. Williams, G. Pertea, A. Mortazavi, G. Kwan, M. J. van Baren, S. L. Salzberg, B. J. Wold and L. Pachter (2010). "Transcript assembly and quantification by RNA-Seq reveals unannotated transcripts and isoform switching during cell differentiation." Nat Biotechnol **28**(5): 511-515.

Wang, Z. Y., C. R. Thornton, M. J. Kershaw, L. Debao and N. J. Talbot (2003). "The glyoxylate cycle is required for temporal regulation of virulence by the plant pathogenic fungus Magnaporthe grisea." Mol Microbiol **47**(6): 1601-1612.

Wu, T. D. and C. K. Watanabe (2005). "GMAP: a genomic mapping and alignment program for mRNA and EST sequences." Bioinformatics **21**(9): 1859-1875.

Xue, C., G. Park, W. Choi, L. Zheng, R. A. Dean and J. R. Xu (2002). "Two novel fungal virulence genes specifically expressed in appressoria of the rice blast fungus." Plant Cell **14**(9): 2107-2119.

Xue, M., J. Yang, Z. Li, S. Hu, N. Yao, R. A. Dean, W. Zhao, M. Shen, H. Zhang, C. Li, L. Liu, L. Cao, X. Xu, Y. Xing, T. Hsiang, Z. Zhang, J. R. Xu and Y. L. Peng (2012). "Comparative analysis of the genomes of two field isolates of the rice blast fungus Magnaporthe oryzae." PLoS Genet **8**(8): e1002869.
